# An experimental study of classical truth logic on multi-propositions consistent and incompatible: Dual-process theories and modal syllogistic of deduction

**DOI:** 10.1371/journal.pone.0299741

**Published:** 2024-07-02

**Authors:** Salma Waheed, Abdul Waheed, Sana Habib

**Affiliations:** 1 School of Psychology, Shaanxi Normal University, Xi’an, China; 2 School of Management, Jiangsu University, Zhenjiang, China; 3 School of Mathematics, Shaanxi Normal University, Xi’an, China; West Pomeranian University of Technology, POLAND

## Abstract

This study looked at a classical truth logic of multi-propositions that is new in some ways: [1] Alethic modalities were mixed with logical consistency and incompatibility in a single plate form, i.e., necessary consistency (NC), possible consistency (PC)/ possible incompatibility (PI) and impossible incompatibility (IPI); [2] multi-propositions were judged by individuals as either NC, PC/PI, or IPI; [3] Four quantifiers; All (∀), No (∼∀), Some (∃), and Some Not (∼∃) of four propositional modes and three shapes (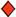, ▱ and 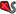) are used to evaluate predictions; and [4] it inspired by multi-propositional of dual-process theories (DPTs) of deduction and modal syllogistic of multi-propositions, from which logicians have derived general hypotheses. HP 1- Individuals will more likely to endorse inferences as PC/PI rather than NC. HP 2: It’s easier to calculate that inference has PC/ PI if it has also NC. Generally, logicians predict more endorsing PC for NC than for PI proposition. HP 3: It’s easier to calculate that inference is not NC if it is also not PC. Generally, logicians predict more PI than IPI proposition endorses as NC. A modal syllogistic as a classical truth logic is presented by multi-propositions (two premises and one inference), each one from four modes has quantifiers such as universal quantifiers and existential quantifier; ∀, ∼∀, ∃, and ∼ ∃. They were evaluated by a single-mental model (Experiment I) and a multi-mental model (Experiment II). Logicians applied the immediate inference task (IIT), evaluation task (ET), and production task (PT) to evaluate three experiments. The results of the experiments suggested that students mostly endorsed PC/PI inferences over NC inferences. Even when logicians divided PC/PI separately as PC and PI, individuals endorsed PC most likely as compared to NC, and PI than IPI. Logicians also highlighted fallacies that were continuously resisted and endorsed when students were asked to judge multi-propositions that had NC. The purpose of this experimental study is to present a glimpse of students’ endorsement of multi-propositions and explain that each individual has a different working memory and intelligence.

## Introduction

A classical truth logic is the core of multi-propositions. These propositions concern the validity of inferences. A logical truth may decide that propositions are possibly either consistent, incompatible or may both. Individual judgment can decide the validity of the inference as to what is necessarily consistent or what is possibly consistent or incompatible. Logicians will resolve their judgments. It’s a logical truth that the shape of the Diamond (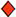) is Rhombus (▱). Logical truths are Formal [1]. Formal logic was first presented by Aristotle in the 1960’s as the theory of modality. Alethic logic has modalities such as Necessary, Possible, and Impossible [2]. These modalities have logically intermingled with consistency and incompatibility as Necessary Consistency (NC), Possible Consistency (PC)/ Possible Incompatibility (PI), and Impossible Incompatibility (IPI) which can be seen in Table 1. Alethic modal operators are usually written as Necessary (☐), Possible (◇) [3], and Impossible (⟢). The formula ◇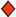 reads and interprets as “it is Possible that Diamond”. These symbols relate with equivalence ☐
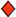
**≡** ¬ ◇¬ 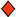, and theoretically predicate further [4].

**Table 1:** Main domain of Alethic Model operators with logical consistency and incompatibility.

Alethic models	Logic	Domain	Reference
Necessary	Consistency	1.“Necessary consistency satisfies all logic”. 2. “the consistency of the definition is necessary and sufficient” 3. “Consistency is considered as necessary”	[5] [6] [7]
Possible	Consistency	1. “Possible consistency used as models for Processing-in-memory operations”. 2. “Number of classifications are possible which are also consistent? In this sense, what in logical” 3. “Possible consistency implied as post system of propositions calculus”.	[8] [9] [10]
Possible	Incompatibility	1.“Interpretation of the infant studies as reflecting representations of incompatible possibilities”. 2. “Incompatibility is primitive. It is after all possible”. 3.“Is it possible to reduce the foundations of logic to the mere concept of incompatibility?”	[2] [11] [12]
Impossible	Incompatibility	1. “Incompatibility problem has been considered: the impossibility intuition”. 2. “logical impossibility as expressed by means of a incompatibility”. 3. “Is it impossible to reduce the foundations of logic to the mere concept of incompatibility?”	[13] [14] [12]

Logical theories contain with theorems’ set all of which is stated logical truth. Classical truth logics are logical theories which theorems are syllogisms [15]. Aristotle has been investigated the concept of syllogisms [16]. A classical truth logic consists of multi-propositions (two premises and one inference). Each of these has Four quantifiers; All (∀), No (~∀), Some (∃), and Some Not (~∃) of four propositional modes and three shapes (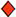), ▱ and 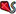) instead of M, P and Q. such example of classical syllogistic logic is:

All 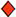 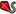


Some ▱ not 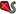


Therefore, some 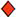 not 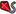


Here, each propositions have a quantifier “all” or “some”. Over the many years, the mental model theory (MMT) has been modified the results [17]. This shows that for coping syllogisms, individuals develop diverse strategies [18]. So, the MMT has been extended to use for more than one quantifier.

The DPTs (MMT and MLT) are prime to solve and evaluate the propositions (Carreira, S., Amado, N., & Jacinto, H. (2020). These dual-process theories of deductive reasoning help the logicians to interpret multi-propositions. As we know that deductive MMT is still worked as a hot issue in academia, hundreds of many experiments reported in new literature see [19], for a review). Still, it’s a curious limitation in all works, further need to investigate. Deductive MLT is rule from logical calculus [20] support to infer modalities. Logicians asked previously; what is necessary inferences from premises, but they did not ask individual about what is necessarily consistent, possibly consistent/ incompatible or impossibly incompatible in multi-propositions. There have been no experiments priors to cover these points in one plate form as logician’s hypothesis that individuals will more likely to endorse inferences as possible consistency/ incompatibility rather than necessary consistency. Logicians describe that DPTs of deduction helps to generate and investigate further hypothesis as discussed below in detail.

In Alethic logic, inferences validity base on truth in deduction, if the multi-propositions are true the premises and inference must be true. Standard logic inference states what is case, but in Alethic modal logic states that what is possible consistency/ incompatibility in case, what is necessary consistency in case, and what is impossible incompatibility. Mostly psychological experiments investigated reasoning of inference about what is the case [21–23], students were asked inference validity, evaluation task (ET) or production task (PT) and problem-based on negations, connectives, if, and, or syllogism. There are very few findings of modal logic reasoning [24, 25] cover these all work in one experimental study.

This experimental study provides support for the validity of students’ cognitive assessments, specifically in the context of evaluating multi-propositions. By demonstrating that students exhibit varying judgments when faced with multi-propositions, the study underscores the complexity of their cognitive processes. Furthermore, the study suggests a positive impact on both working memory and intelligence, indicating that engaging with and evaluating multi-propositions can contribute to the enhancement of these cognitive functions among students. In essence, the findings highlight the significance of considering diverse cognitive tasks (IIT, PT, and ET), such as judging multi-propositions, in educational settings to foster cognitive development and intelligence. Moreover, this study has been inspired by multi-propositional DPTs of deduction and modal syllogistics of multi-propositions, from which logicians have derived general hypotheses.

### Multi-propositional DPTs of deduction

The DPTs of deduction are two main competing theory; MMT and MLT. These theories have been got success over the last few years [26]. This study has been implemented this dual-process in multi-propositions. Here is the brief intro of these theories; MLT was promoted by Martin Braine and David O’Brien, as well as Lance Rips and others [27]. The central claim of the theory is as follows: Human reasoning utilizes mental representations similar to true propositions. In deductive reasoning, the reasoner manipulates these multi-propositions by applying the reasoning’s rules as syntax corresponding to logical rules. Different versions of the theory of mental logic differ in which rules are used in deductive reasoning. However, they generally claim that these rules are similar to those found in the true deductive formulas of formal logic. Some of these rules involve the use of hypothesis as a classical syllogistic logic. For example;

All △ ◁

Some ◁ not ▷

Therefore, some △ not ▷

**Fig 1:**
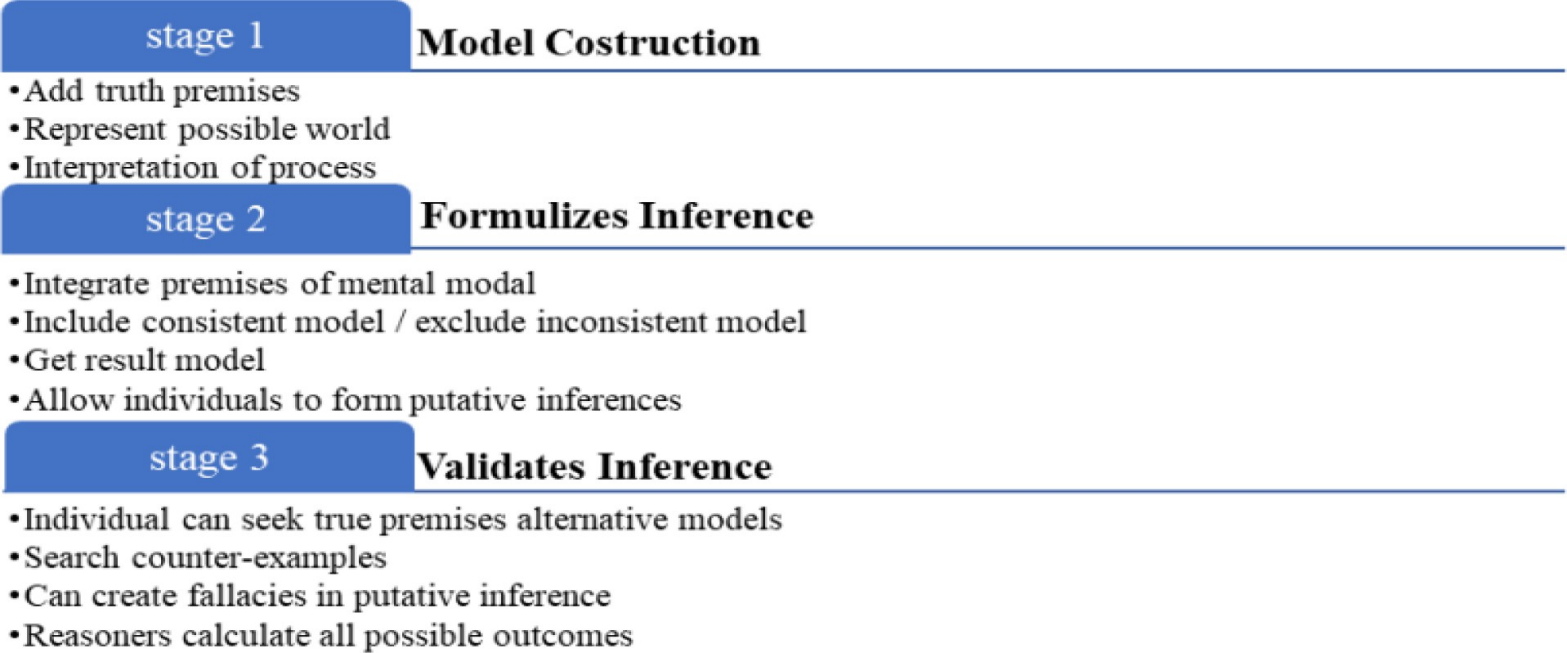
Three stages of MMT.

Here, △ denoted M, ◁ denoted P, and ▷ denoted Q. Some fallacies were firmly endorsed. MLT explains fallacies in deductive reasoning tasks by alluring to the difficulty of applying specific rules and the need to apply more than one rule in a given task. This theory also called Rule-based theory [18, 28]. It drives from true logical deduction, assumes rules of multi-propositions. This study choice MLT due to two intentions. First, MLT theorists have mostly restricted their conceptual and experimental tasks to the find the propositional inference, very brief to affirm about syllogistic deduction (Rips, 1994, for few debates of quantifier inference). Second, the rare published theories only provide rules for valid inferences and thus explain necessary rather than possible inferences. We concede significance of how to extend MLT to account for possible consistent/incompatible inferences of the general discussion. Whereas; the MMT developed by [29]. This theory expresses the concept of logical reasoning as the process that develops three stages can be shown in Fig 1.

Each individual has different working memory and intelligence, they judge multi-propositions differently. Development of MMT relates with validity of inference from premises. In facts, individuals can do many logical fallacies on the given task and exhibit logical errors. Human rationality has led a big debate. However, mostly psychologists whose work relate with this area are agreed that untrained students do perform some basic deductions: such as they can differ valid inference from falsity but some cannot. Still yet this debate unresolved between two theoretical perspectives. This study debate focuses on whether deduction of true logic achieves answers through MLT rules and MMT models. Inspired by [17], this study present a classical truth logic presenting production task (PT) methodology. We used multi-propositional syllogistic modal logic with all possible premises (see below). Individuals were enquired to derive inference from given premises. We analyzed that these multi-propositions of multi-models have high rate of impossible incompatibilities. So, this study presents a principle that multi-propositions of multi-models place stain on working memory and intelligence. In particular, individuals commit fallacies due to flop of find a counter-example in MMT at Stage 3, however, such models exist. Hence, the DPTs of deductions predicate the concern of modal syllogistics of multi-propositions are discuss bellow.

### Modal syllogistic of multi-propositions

The modal syllogistic is made distinction between Alethic logic with consistency and incompatibility such as, necessary consistency (NC), possible consistency (PC), and possible incompatibility (PI) impossible incompatibility (IPI). If the proposition is Alethic, it is true factual; if the proposition has NC, it must be true, if the proposition has PC/ PI, it may be true, and if the proposition has IPI, it must not be true. To consider these concepts MLT following rules of multi-propositions are as under;

1. All the cagers are tall,
  Abdul Jabber is a cager,
  Therefore, it’s *necessary* that Abdul Jabber is tall.   Necessary Consistency (NC)
2. All the cagers are tall,
  Abdul Jabber is a cager,
  Therefore, it’s *possible* that Abdul Jabber is tall.   Possible Consistency (PC)
3. All the cagers are tall,
  Abdul Jabber is a cager,
  Therefore, it’s *possible* that Abdul Jabber is not tall.   Possible Incompatibility (PI)
4. All the cagers are tall,
  Abdul Jabber is a cager,
  Therefore, it’s *impossible* that Abdul Jabber is not tall.   Impossible Incompatibility (IPI)

MLT rule 1 NC has a modus ponens inferences that conclude necessity state, their propositions are true, indeed it’s *necessary* that Abdul Jabber is tall, and this inference is valid. Rule 2 PC inferences are also valid, however as compare with strong inference NC (it’s *necessary* that Abdul Jabber is tall.), PC (it’s *possible* that Abdul Jabber is tall.) would be invalid, why because their propositions have consistency with the situation where there are cagers who are not tall, so Abdul Jabber may be a cager who is not tall. Rule 3 PI have invalid as NC shows, Abdul Jabber is necessarily tall given premise and rule 4 it’s *impossible* that Abdul Jabber is not tall is completely invalid. Logicians refer these states as necessary consistency (NC), possible consistency (PC) or possible incompatibility (PI) and impossible incompatibility (IPI). Deductive reasoning standard guidelines require individuals to mark decisions about the validity of Alethic inferences. In MLT, if individuals are enquired to decide whether A has NC, where A is a proposition, then they must be endorsed the NC inferences and reject the PC/PI and the IPI. Conversely, if persons are enquired to decide whether H has PC/PI, where H is a proposition, then they must be endorsed the NC inferences as well as the PC/PI and reject the IPI. In the normal psychological study of MLT reasoning, the ability to distinguish between PC/PI and IPI inferences is neglected.

The theory first: Individuals judge whether multi-propositions are necessarily consistent? Individual judgement can decide the validity of inference as either necessarily consistent, possibly consistent/incompatible or impossibly incompatible.

The theory second: whether their judgment, correct?

Logicians asked from individuals to decide whether multi-propositions are NC, where multi-propositions are llsertions, and they must endorse the NC inferences and reject the PC/ PI and IPI. Conversely, if individuals are asked to decide whether multi-propositions are PC/ PI, then they can endorse NC inferences as well as PC/ PI inferences and only reject IPI inferences. The ability to distinguish PC/ PI from IPI inferences is ignored in usual psychological investigates on MLT reasoning. Considering the three general phases of MMT have defined prior. Logicians have constructed propositions’ single-model that supports tentative inferences during first two phases. These inferences can report as far as PC/PI without any further logical reasoning. Inferences validity phase (finding counter-examples) comes into play only when person is enquired to prove that the hypothesized inference has NC. Therefore, according to mental model (MM), PC/PI inference must be easier than NC.

Therefore, our first hypothesis (HP) is as follows:

**HP 1:** Individuals will more likely to endorse inferences as PC/PI rather than NC.

The MMT is considered three propositions which help to clarify further hypotheses.

NC proposition: the inference is true in all MMs of the premises.

PC/ PI proposition: the inference is true in at least one MM of premise.

IPI proposition: the inference is true in no MM of premise.

After this analysis, Logicians drive two more hypotheses on the basis of these assumptions:

**HP 2:** It’s easier to calculate that inference has PC/ PI if it has also NC. Generally, logicians predict more endorsing PC for NC than for PI proposition.
**HP 3:** It’s easier to calculate that inference is not NC if it is also not PC. Generally, logicians predict more PI than IPI proposition endorses as NC.

HP 2 explains only to follow NC as discover MM of premise that supports the inference inferring that it is PC/ PI. On NC propositions, all MMs of premises support the inference, while on PC/PI proposition, at least one MM of premise does not. Thus, in the latter case, it is *possible* for the Logicians to first consider the MM of premise that does not support the inference. HP 3 follows closely because no inference is required when at least one MM of premise does not support it. On IPI proposition, there are no antecedent MMs of premises to support the inference, whereas on PI proposition there is at least one supporting inference. Therefore, on the latter propositions, they are likely to first think of the MM of premise that supports the inference.

Logicians reports three experiments to design the test, Experiment I and II addresses the theories hypothesizes, and predictions, and the Experiment III defines the problems that arise from first two experiments. In Experiment I, logicians tested the first theory: Individuals judge whether multi-propositions of multi-models are necessarily consistent? and the above hypothesis by using simple task of a classical truth logic. In this task, logicians involve to give individuals single propositional premise of syllogism and suggest them to make two or more different propositional inferences, and this task is called “immediate inference task (IIT)” [30]. So, experiment II tested the second theory: whether their judgment, correct? And given hypothesis. This study involves full syllogism logical reasoning, ask individual to define multi-propositional each pair of *possible* inferences with regarding each *possible* premises through valid NC judgments. These studies include groups of people require to develop judgments of NC as well as PC/ PI inference. Both studies have designed together with running on same individuals in similar sessions. However, due to the difference in the size of the two study experiments balancing the order of studies does not appear to be appropriate. Nor is it advisable to have the same individuals make judgments about NC and PC/ PI. The subsequent procedure is as proceeds. people were separated into necessary (□) and possible (**◊)** groups and completed the brief tasks of study experiment I with an appropriate rule form. Each individual then executed the big task essential in study experiment II performing the identical form of rule assigned to them in study experiment I. These groups were also subcategories in study experiment II affirming to obtain terms in the inferences they were described (perceive the methods select of study experiment II for details). Logicians have also reported experiment III that is replicated some imported findings of experiment II by using quite smaller subsets of a classical truth logic with simple method of multi-propositions presentation. Experiment III is also provided a check of the Experiment II’s results that are not influence with Experiment I’s results.

## Methodology

### Experiment I

A modal syllogistic as a classical truth logic is presented by multi-propositions (two premises and one inference), each one from four modes have quantifiers such as universal quantifiers and existential quantifier; ∀, ~∀, ∃, and~ ∃.

 ∀ Universal Affirm All M P

 ~∀ Universal deny No M P

 ∃ Existential Affirm Some M P

 ~∃ Existential Deny Some M Not P

In Experiment I, Logicians only examined the immediate inferences task (IIT), individuals can draw multi-propositions as one proposition is the premise and the other one is the inference. As the order of the terms can be reversed, for each of the 4 *possible* premises, there are 7 *possible* inferences (repetitive premises have been excluded) to consider.

Syllogistic immediate inferences’ propositions have grouped them into 3 types and established following sub-types:

NC: Given premises are true so that inferences must be true. Among 28 multi-propositions 6 fall in this type.

PC/ PI: Given premises are true so that inferences may be true. Among 28 multi-propositions 12 fall in this type.

IPI: Given premises are true so that inferences may not be true. Among 28 multi-propositions 10 fall in this type.

To note that any inference which has logically PC that is NC as well and the proposition logically classify as PC/ PI have in fact those in which inference has PC and not NC.

By demonstrating this concept, logicians have considered an example in which what can infer from proposition “All 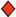 ▱” here, Diamond (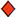) denotes M and Rhombus (▱) denotes P. Each *possible* inference is as follow:

- No 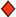 ▱
- Some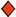 ▱
- Some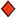 not ▱
- All ▱ 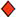
- No ▱ 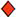
- Some ▱ 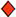
- Some ▱ not 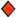

Inferences a and f are NC; d and g are PC/ PI (but not NC), whereas a, c and e are IPI. However, if the individuals were instructed to judge whether all inferences were NC given the premises, b and f would be yes as correct answer and no answer would be for others. Same as for PC/PI, correct answer yes would be for b, d, f and g and no answer for a, c and e.

In Experiment I, rules were presented to the individuals which were either NC or PC/PI with immediate inference propositions. That helps logicians to test hypothesizes those have identified initially. Here mention that in this experiment, there is an inevitable confusion among the type of rules (NC or PC/PI) and the type of response that constitutes the correct answer. For example, since more than two different inferences are actually PC/PI than NC, any general bias in accepting inferences will conduct more accurate responses in the ◊ group than in the □ group. Although this does not bring logicians with the propositional testing of the above-mentioned hypothesis, which in any case are based on accepted inferences rather than correct decisions. Here mention also that HP2 and HP3 together are involved with intragroup comparisons, where the rule type remains the same.

### Experimental method

#### Students

We determined classical sample size. This experiment was Between-Group design (see Table 2). Sample size was 86 but 6 students’ reposes were bizarre, so, we excluded those responses. 80 university students (50 men. 30 women, age average: range 23–30 years) from one University of Pakistan have taken part in this experiment. All are Urdu speakers but their study medium is in English. They did not study logic before. To ensure adherence to ethical principles, we got official written approval from the Ethics Committee of Shaanxi Normal University, School of Psychology. Informed written consent was obtained from all study participants, who claimed their understanding of the study’s purposes, and the respondents were assured that their identities and responses were kept confidential which would be maintained in the best interests of academic integrity and every possible manner. Additionally, they were informed of the importance of the study, its possible implications, their role as interviewees, and that no compensation would be offered and they would like to participate in the study voluntarily. Respondents were assured that they could stop responding and withdraw from the interview at any time. The data collected will be used only to suggest mechanisms for abating the impostor’s feelings through academic outcomes.

**Table 2:** Between-subjects factors of Experiment I.

		Value Label	N
GROUPS	1	□ group	28
	2	◊ group	28
PROPOSITIONS	1	NC	12
	2	PC/PI	24
	3	IPI	20

#### Design & material

Students were divided into two experimental groups; *necessity* (□) group and *possibility* (◊) group 40 in each. Each group has their own rules and they evaluate the 28 multi-propositions of IIT. All the propositions have described independently and they generated random task order (see Table 2). Both the groups and Propositions are independent variables and the student’s endorsement of inferences are dependent variables in this Experiment. The students firstly performed Experiment I and then they would proceed to carry out the Experiment II task at the same scenario, explained below.

Task was performed in the computer lab of the university. Online questionnaire was displayed via google forms [31]. All the students performed the task at the same time. Separate computer desktops were used for each student base on 28 multi-propositions with the layout such as:

Given that

Some Rhombus (▱) are Diamonds (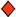).

Is it *necessary* that

Some Diamonds (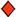) are not-Rhombus (▱)

Above is the example of □ group. Same as ◊ group read “Is it *possible* that”. Beside are these boxes base on *Yes* and *No* response. Students were indicated their decision by using mouse to click on yes or no box.

This program was randomly assigned the shape to sentence excluding ∃ and ~∃.

#### Procedure

Students were divided into two groups in computer lab of the university as there several computers run on same google form. Each individual had his own desktop and he did his work individually at his admit space.

Students were solely allotted to the experimental condition after enter the computer lab.

Following rules have been given by the students:

- Each student has randomly allocated one computer desk.
- Instructor told them to take maximum time to finish the task.
- Students must be taken part in the experiment individually.
- Instructor told them their information would be personal.
- They had authority to depart at all time.

The forms based on the rule of Experiment I were sooner present on the computer terminal of students. They were enquired to study all manuscript and would be started as soon as they can. Instructor told them that this experiment was designed to know about how do individuals solve multi-propositions of true logical reasoning? Instructor also told the students that if the inference follows the premises click on yes box. Each student received 28 multi-propositions and they were answered them randomly. Both groups’ answers were covered by the google form and saved into the disk.

#### Results and discussion

**Fig 2:**
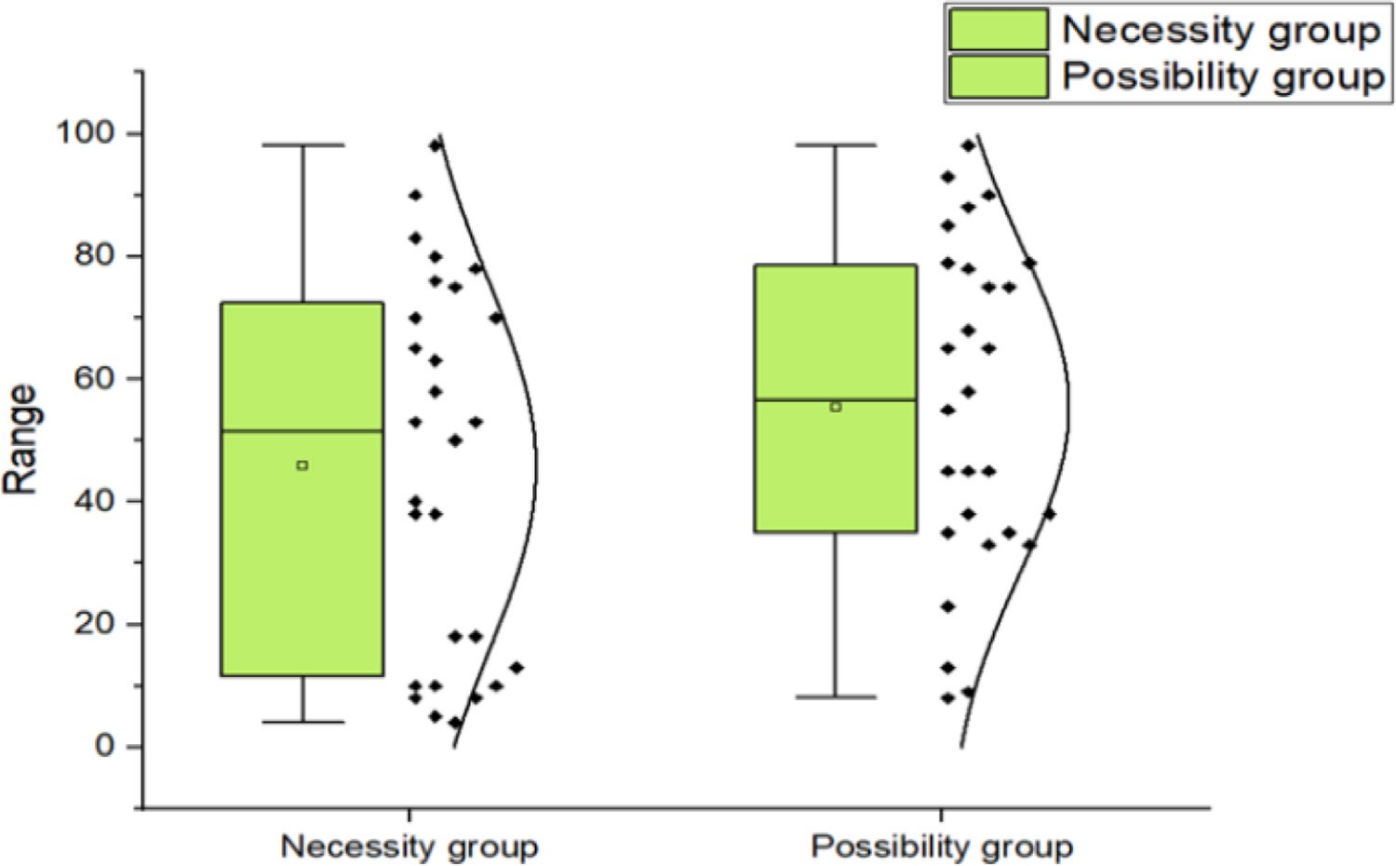
Inferences percentage of immediate inference task (IIT) in Experiment I, with rules of □ and ◊ groups.

The experiment contains the sets of datasets that can be investigated for many conceptual aims rather than those logicians have here for spatial description. Therefore, an entire enlist of the percentage of inferences agreed by all groups for all premises is given in S1 Appendix. Logicians have focused their analysis here on three modalities with consistency and incompatibilities of multi-propositions, identified a priori as inferences, that are NC (n = 6), PC/PI (n = 12), or IPI (n = 10) given the multi-propositions. Fig 2 shows the average percentage approval of inferences in all sets for the two rules’ groups. Here mention that more inferences endorse in ◊ group, and that the order of approval for multi-propositions types NC > PC/PI > IPI in both rule formats. The latter tendency is to be expected, not only for DPTs, but for any interpretation that takes into account the important factor of deductive power in classical truth logics.

As we calculated in both groups that the Average Means percentage of PI/PC (μ = 65.08, SD = 30.095, SE = 8.688, P < .5 and μ = 68.58, SD = 21.047, SE = 6.076, P < .5) is greater than NC (μ = 56.50, SD = 16.453, SE = 6.717, P < .5 and μ = 60, SD = 14.895, SE = 6.081, P < .5). As we strongly accepted our hypothesis that HP1: Individuals will more likely to endorse inferences as PC/PI rather than NC. Descriptive statistics are as under (see Table 3).

**Table 3:** Descriptive statistics of Experiment I.

		N	Mean	Std. Deviation	Std. Error	95% Confidence Interval for Mean		Minimum	Maximum
						Lower Bound	Upper Bound		
◊ group	NC	6	56.50	16.453	6.717	39.23	73.77	35	75
	PC/PI	12	65.08	30.095	8.688	45.96	84.20	13	98
	IPI	10	44.70	34.192	10.813	20.24	69.16	8	98
	Total	28	55.96	29.881	5.647	44.38	67.55	8	98
□ group	NC	6	60.67	14.895	6.081	45.04	76.30	38	78
	PC/PI	12	68.58	21.047	6.076	55.21	81.96	18	90
	IPI	10	13.60	14.462	4.573	3.25	23.95	4	53
	Total	28	47.25	30.870	5.834	35.28	59.22	4	90

A 2*3 ANOVA and post hoc Test (PHT) were performed to exam the effect of Between-subjects’ groups i.e., □ group and ◊ group, and Within- subjects inferences set i.e., NC vs PC/PI vs IPI. we analyzed the depended variable as overall means percentages of inferences that all the students’ endorsement. Each proposition was calculated as we were provided info in three sets to the ANOVA (see Table 4).

**Table 4:** Means percentage of inferences between groups and within groups subject ANOVA.

		Sum of Squares	df	Mean Square	F	Sig.
◊ group	Between Groups	2268.448	2	1134.224	1.298	.291
	Within Groups	21838.517	25	873.541		
	Total	24106.964	27			
□ group	Between Groups	17864.600	2	8932.300	28.394	.000
	Within Groups	7864.650	25	314.586		
	Total	25729.250	27			

All the results showed that ◊ group rules significantly endorsed student’s judgment estimation possibly true as ◊ group Between Group significant rate is .291 rather than □ group significant rate is Zero. Hence, the main effects of the results accept main hypothesis HP1, defined initially. This interaction also reflected the high difference among two groups for inference that have *possibility*. Moreover, ◊ Group had also a very consequential effect as considering greater recognition under the ◊ group F (l, 27) = 55.96, SE = 5.647, p < .05.

This interaction seemed to reflect a larger difference in the inferences that could be drawn between the two groups. This is not surprising, since the correct answer to these inferences is different among both groups: “Yes” for the ◊ group and “No” for the □ group. Subsequent analyzes were performed to evaluate HP2 and HP3 taken from the DPTs. As a retrieval, HP 2: It’s easier to calculate that inference has PC/ PI if it has also NC. Generally, logicians predict more endorsing PC for NC than for PI proposition.

Such predicted hypothesis rationale is the only one requirement; identify MM which supports the inference to be sure that it is PC/ PI. On NC propositions, all MMs of the propositions support the inference, and on PC/ PI proposition, at least one of the MM does not (PI proposition). So, it would be ease to find an endorsing MM on the NC proposition. This hypothesis was tested using one-tailed relative group t-test, comparison with given sets PC/ PI and NC propositions for the □ group rule. This hypothesis was significantly agreed, t (39) = 5.44, p < .001. HP3 as follows It’s easier to calculate that inference is not NC if it is also not PC. Generally, logicians predict more PI than IPI proposition endorses as NC. Logicians predict more PI than IPI in the □ group. This predicted hypothesis only rationale one requirement; identify one MM of the propositions PI that contain no inference PC to determine the inference. This is easier on IPI proposition, where no proposition model supports the inference, than on PI proposition, where at ease one MM would endorse it. This HP3 was evaluated by “one-tailed t-test” to compare accepting rates for PI and IPI proposition under the □ group rule and was additionally strongly accepted, F (39) = 16.91, p’s < .001. Additionally, we concluded in Table 5, in both groups; PC/PI with NC highly significant (sig. = .832, .650) as compare with IPI (sig. = .260,.000). So, we strongly accept our HP2, HP3 and predictions. Logicians’ analysis the inferences drawn from the IIT of Experiment I strongly supports all the main hypothesizes and predictions. Another aspect of the occurrence data is also worth noting.

**Table 5:** Multiple comparisons between groups and within subject inferences. Tukey HSD.

Dependent Variable	(I) NC vs PI/PC vs IPI	(J) NC vs PI/PC vs IPI	Mean Difference (I-J)	Std. Error	Sig.	95% Confidence Interval	
						Lower Bound	Upper Bound
◊ group	NC	PC/PI	-8.583	14.778	.832	-45.39	28.23
		IPI	11.800	15.263	.723	-26.22	49.82
	PC/PI	NC	8.583	14.778	.832	-28.23	45.39
		IPI	20.383	12.655	.260	-11.14	51.90
	IPI	NC	-11.800	15.263	.723	-49.82	26.22
		PC/PI	-20.383	12.655	.260	-51.90	11.14
□ group	NC	PC/PI	-7.917	8.868	.650	-30.01	14.17
		IPI	47.067*	9.159	.000	24.25	69.88
	PC/PI	NC	7.917	8.868	.650	-14.17	30.01
		IPI	54.983*	7.594	.000	36.07	73.90
	IPI	NC	-47.067*	9.159	.000	-69.88	-24.25
		PC/PI	-54.983*	7.594	.000	-73.90	-36.07

*The mean difference is significant at the 0.05 level.

**Fig 3:**
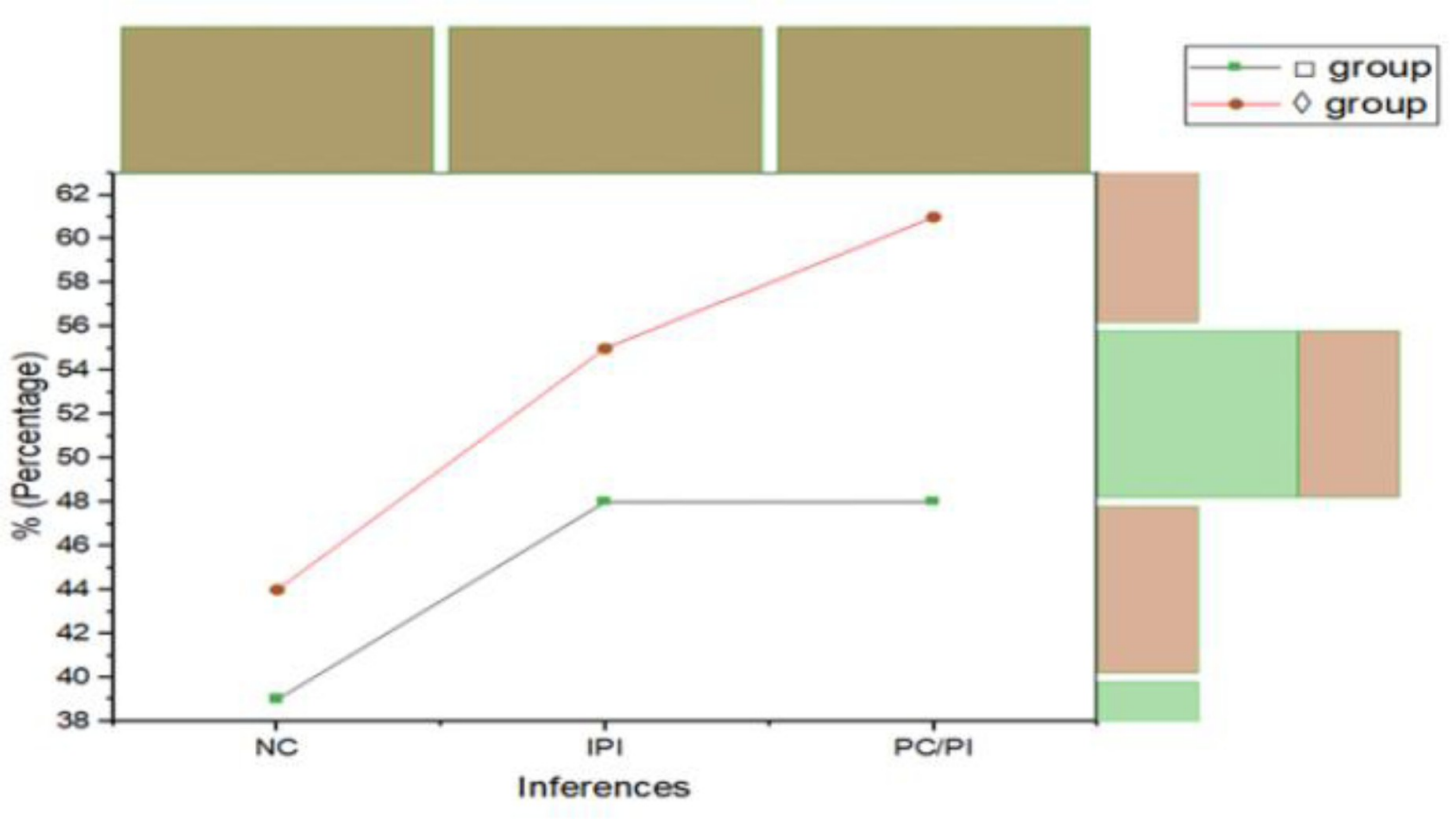
Means percentage of inferences between both groups.

Although PC/PI propositions were recognized almost same frequently than NC and IPI propositions in both rules in groups, the data under the propositions of □ group appeared to serve multi-modal distribution. 12 PC/ PI propositions, accepted percentage was 48%, same as 10 IPI propositions percentage was 48 and of the 6 NC percentage of inference was 39%. whereas, in ◊ group, PC/ PI inference percentage 61%, NC percentage 44% and percentage of IPI propositions were 55% (see Fig 3). Thus, the results were shown that logicians often did not look for counter-examples, indeed when ruled to develop the □ group findings.

The data are compatible with the hypothesis that few PC/ PI propositions propose an initial MM that endorses the inferences—inducing to the endorsement of fallacies—while rest one do not. logicians back to this proposition when they discuss the consequences of Experiment II and provide the reason for Experiment III, all of which involved modal syllogism.

### Experiment II

Experiment II defined this study in detail as report in Experiment I, Logicians firstly briefly discussed the classical truth logic as a modal syllogistic logic. A syllogism has two terminal shapes, which they call Diamond (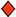) and kite (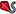) (here Diamond shape donated M and Kite shape donated Q)- in the order mentioned in the assertions—and a midst or connecting atom, which they call Rhombus (▱) (Here Rhombus shape donated P). The order of inferences can be 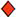
- ▱ or ▱-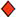. premises or inferences can take each of the four forms of modes were discussed previously and were explored in the IIT of Experiment I. Such as;

All 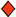 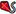


Some ▱ not 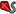


Therefore, some 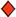 not 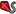


A classical truth logic has distinguished the four shapes of the syllogism that was given by order of study in the assertions with endorsement to the order in inference. A classical truth logic is also explained as only way to establish propositional sequence. In this study, logicians have wanted to consider all possible ways to present these propositions of inference. Therefore, they have followed the Johnson-Laird and Bara [17] defined figure by their self-generating shapes without regard to the order of premises to inferences. There are four possible shapes:

Shape A  
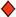
**- ▱**

      ▱ -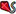


Shape B  **▱ -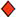
**

      
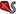
- ▱

Shape C  
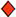
**- ▱**

      
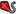
- ▱

Shape D  **▱—
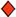
**

      ▱ - 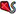


Here 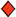 denotes M, ▱ denotes P, and 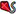
denotes Q. This experiment has used Evaluation Task (ET) and each of above shape can be defined inferences of either 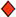

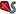
or 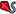

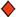
order. PC/ PI propositions’ number in the ET increases significantly, since the two PC/ PI order of inferences must be compound by the four *possible* modes. This resulted in five hundred twelve particular propositions. All of these were evaluated in Experiment II. However, to keep the task within reasonable bounds, students assessed 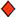

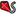
or 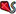

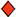
order, but not both. Furthermore, they assessed 4 inferences on the similar desktop for a given pair of premises, so they efficiently assessed two hundred fifty-six syllogistic propositions, presented on 40 separate desktops.

Many syllogistic literatures of reasoning have been detected in the previous academia [18, 32, 33]. Recently, the main development of syllogistic reasoning is modal syllogistic logic [34]. As here mention earlier, with regard to the deductive approach, Rips [20] provided a preliminary account of reasoning with quantifiers in the tradition of MLT. MMT theorists have described syllogistic reasoning in detail over the years and have implemented it in working computer programs. The linguistic inference model, which is likewise model-based but diverges significantly from the theory put forward in several significant ways, was significantly improved by Polk and Newell [35]. Previously, we mentioned that the goal of this study is to verify general DPTs hypothesizes that are independent of implementation details. We examined the three hypotheses (HP I, and HP 2,) about the distinction between PC/ PI and NC inferences that were evaluated on the simple IIT in Experiment I using this Experiment as well.

### Experimental method

#### Students

We determined classical sample size. This experiment was Between-Group design (see Table 6). 80 participants were same who performed experiment 1. Now, total 110 university students (65 men. 45 women, age average: range 23–30 years) from one university of Pakistan have taken part in this experiment. All are Urdu speakers but their study medium is in English. They did not study logic before. To ensure adherence to ethical principles, we got official written approval from the Ethics Committee of Shaanxi Normal University, School of Psychology. Informed written consent was obtained from all study participants, who claimed their understanding of the study’s purposes, and the respondents were assured that their identities and responses were kept confidential which would be maintained in the best interests of academic integrity and every possible manner. Additionally, they were informed of the importance of the study, its possible implications, their role as interviewees, and that no compensation would be offered and they would like to participate in the study voluntarily. Respondents were assured that they could stop responding and withdraw from the interview at any time. The data collected will be used only to suggest mechanisms for abating the impostor’s feelings through academic outcomes.

**Table 6:** Between-subjects factors of Experiment II.

		Value Label	N
Groups	1	□ group-MQ	128
	2	◊ group- MQ	128
Shapes	1	Shape A	128
	2	Shape B	128
	3	Shape C	128
	4	Shape D	128
Inferences	1	NC	48
	2	PC/PI	416
	3	IPI	48

M denotes 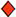, and Q denotes
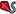

#### Design & material

Total 110 students (55 in each group) performed task in four subdivided experimental orders. Students got the similar type of experiment’s rule (□ and ◊ group) as in Experiment 1, however they were subgroup accorded to how they accepted inferences assessed in 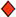

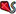
or 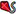

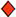
orders.

Each student was given 55 independent computer desktops, one for all probable propositional pairs. For all pair, we were indicated how all of the four probable inferences were rules of □ and ◊ group. The four possible inferences are for each of the four modes, and the order (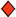

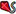
or 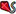

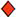
) depends on the sub-groups to which the student was assigned (see Table 6).

Multi-propositions were given in same pattern forms as in Experiment 1 except, two propositions, and four inferences evaluating now. Such as a desktop layout follows under;

Given that

Some △ are ◁

No ◁ are ▷

Is it Necessary that

Yes No

All △ are ▷ □ □

Some △ are ▷□ □

No △ are ▷ □ □

Some △ are not ▷ □ □

Here, Equilateral Triangle (△) denoted as A, left Triangle (◁) denoted as B, and right Triangle (▷) denoted as C. Above were the examples of □ group—P R group. Students in ◊ group- R P group were given same as propositions “Is it possible that…” and those whom were R P group shown inferences reversed i.e., All ▷ are △, and so on. Shapes were given randomly with independent classical truth logic form for the program of each premise. The order of four inferences type as modes were fixed above as ∀, 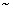
∀, ∃, and 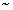
∃ for all propositions.

#### Procedure

Students from Experiment I also participated in this experimental task. They have been told that there would be another series of classical truth logic of multi-propositions, but in this condition, there would be double premises, and we would ask, if the propositions have truth, to judge either subsequent propositions are compulsory true (□ group) or probably true (◊ group). Students were revealed examples of desktop layouts and indicated their responses by clicking "yes/ no" boxes next to all of the propositions: They had authority to decide on the propositions in any answer, but that the answers could not be changed once they were made. A box on the desktop was filled whenever a student clicked inside of it. A box with the title "Click here for the next propositions" appeared when all four responses had been given here for the next problem" appeared when all four responses had been given. Students could take breaks as needed in between the lengthy sequence of multi-propositions and work at their own pace. The application stored the replies and latencies to disk along with the responses. The distribution of the 55 desktop computers to the students was independently random.

#### Results & discussion

**Fig 4:**
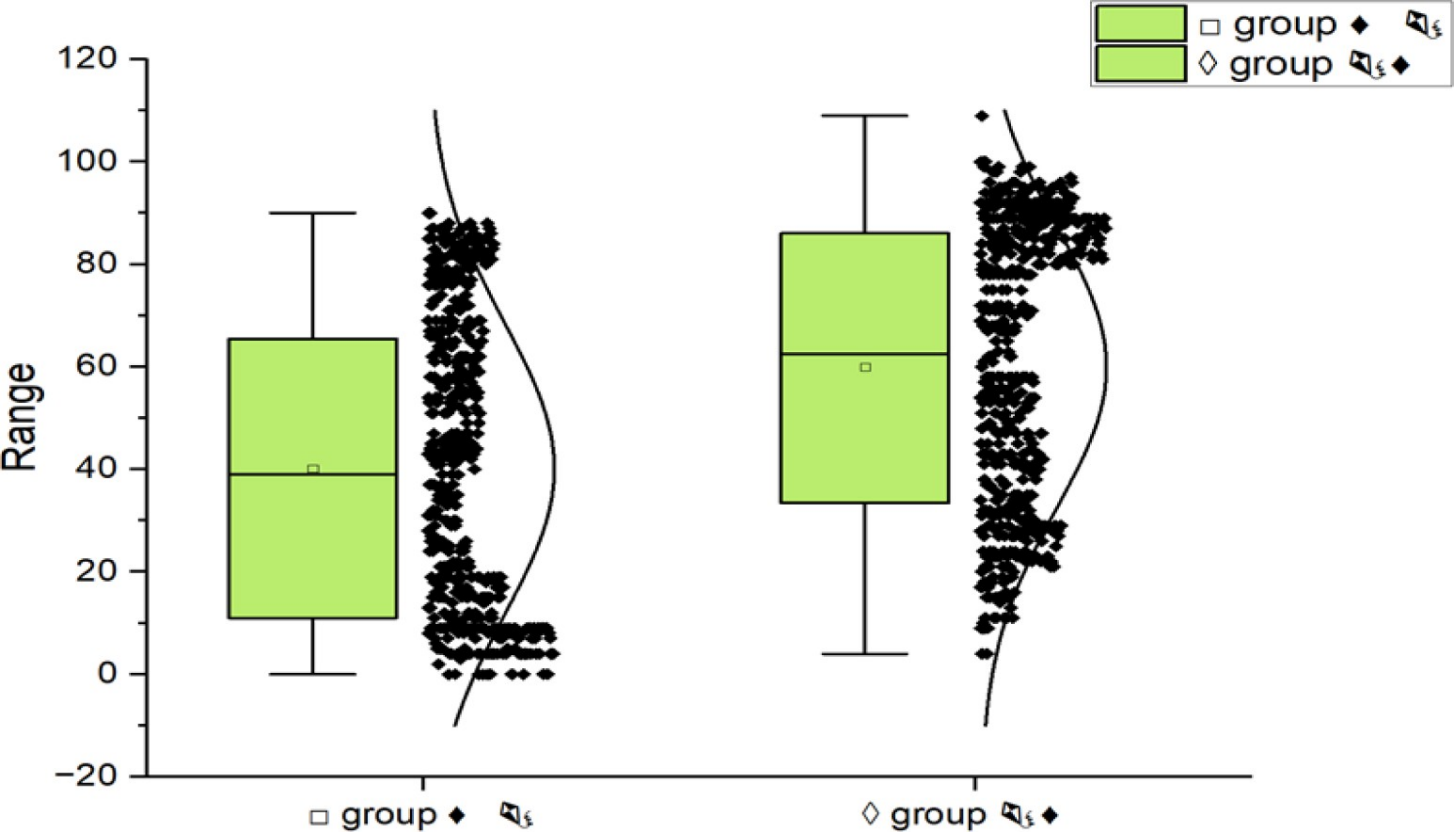
Inferences percentage of A classical logic syllogism in Experiment II, with rules of □ and ◊ groups (♦ 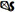
or 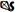 ♦).

S2 Appendix contains a list of response frequencies for each particular multi-proposition. Each student rated 256 premises-inferences combinations, 24 of which were NC (inference must follow premises), 208 of which were PC/PI (inference could follow from premises), and 24 of which were IPI (inference cannot follow true from premises). These figures reflect the fact that if premises exist, given that students, we suppose that universal propositions entail the existential of the convenient students and that "some" is considered to mean "at least some, and potentially all.", such as, support an inference of the form " All ▷ △," they also support "Some ▷ △" and "Some △ ▷." The average means acceptance rate for all proposition type in all rule group is indicated in Fig 4 and is the average of the
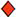

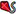
 and 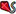

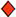
subgroups (this has no overall influence).

A 2*4*3 ANOVA was performed again on the shared information from all students and conducted information’s about all the three propositional types.

As we calculated in both groups and sub-groups that the Average Means percentage of PI/PC is greater than NC. As we strongly accepted our hypothesis that HP1. The results were almost same tendencies shown in Experiment 1, however, the ANOVA was used here that revealed similar patterns showed a substantial main impact of rule groups, showing that □ group
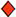

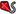
 rules led to better acceptance rates (SS = 16.866, df = 82, MSE = .206, η_p_^2^ = .240) see Table 7.

**Table 7:** Tests of between-subjects effects in Experiment II.

Dependent Variable: NC vs PC/PI vs IPI						
Source	Type III Sum of Squares	df	Mean Square	F	Sig.	Partial Eta Squared
Corrected Model	42.650^a^	167	.255	1.647	.000	.444
Intercept	377.661	1	377.661	2435.140	.000	.876
◊ group Q M	21.332	85	.251	1.618	.001	.286
**□** group M Q	16.866	82	.206	1.326	.044	.240
Error	53.350	344	.155			
Total	2144.000	512				
Corrected Total	96.000	511				
a. R Squared = .444 (Adjusted R Squared = .174)						

M denotes 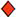, and Q denotes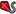.

Additionally, there was a substantial main influence of proposition type that reflected the hierarchy of NC > PC/PI > IPI as independent variables that depend on both the groups and sub groups rate. The interaction among these two factors was strong, just like in Experiment I: It can be shown from that rule had the greatest impact on propositions of the PC/PI type. On the other hand, the sequence of the inferences had no impact. HP1 has confirmed the main impact in ANOVA test, however, HP2 and HP3 also required follow up tasks is in Experiment I. One- tailed t tests groups related was assessed with separated tests for 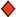

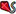
 and 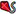

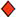
 sub-groups (degree of freedom = 167). HP2 that students given ◊ group rules will admit more NC than PC/PI inference. It’s confirmed for both 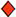

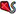
 (F’s = 5.93, p’s< .05) sub-groups.HP3 revealed that students given □ group rules will admit more PI than IPI inference. That confirmed both 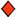

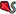
 (t = 11.4, p < .05) and 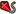

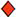
 (F’s = 8.6, p’s < .05) sub-groups.

In classical truth logic of modal syllogism, ET is used in this experiment to look at a few additional interesting multi-propositions in DPTs. According to the MM theory, individuals are less likely to correctly infer inferences from syllogistic propositions that suit several MMs than they are from those that only fit one MM. The key idea here is that any propositional MM will support an inference if it is true, as is the case with their required inquiry. Therefore, there shouldn’t be a modification in performance among single-model and multi-model propositions if individual just considers one MM of the proposition. On the other hand, individuals should be more challenging at deduction if they try to reason establishing an inference in all MMs of a proposition. Logicians admitted that these hypothesizes have not been tested before on a syllogism ET, although Johnson-Laird and Bara [17] provided evidence for it using as production task (PT). To reduce comparison with their studies, we excluded validity of syllogisms into weak inferences from this investigation—such as, those to be evaluated that inference "some 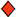 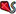
" despite the inference "all 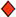 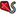
" that was supported by propositions. Such inferences rarely arise in PT. 256 propositions posed to each student, only 36 were valid and led to strong inferences. For these subgroups, logicians investigated the modification in accepting rates among propositions multi-propositions of single-model and multi-model. On the basis of the □ group rules, they would suppose a trend same to that tested by [17], namely, a reduced acceptance of valid inferences for multi-propositions of multi-models. However, under the ◊ group rules, such tendencies shouldn’t be discovered since in this situation, it’s simply NC to find a model that supports the inferences of the students to prove the answer yes. The patterns in their data support the hypothesis. 18 of the 36 propositions fall into the categories of single-models and multi-models, respectively, according to Johnson-Laird and Bara. When given □ group rules, 82% of propositions with a single-model had their results accepted as opposed to 69% of propositions with multi-models. This tendency was less pronounced under ◊ group rules, with 89% of single-model of multi-propositions accepted vs 78% of multi-model of multi-propositions, as was to be expected. For all students, they calculated the modification in accepting rates of single-model and multi-model of multi-propositions in order to assess the relevance of this interaction. One tailed “between-groups” t-test was used to compare these difference scores between the 55 individuals who received □ group rules and the 55 students in the ◊ group rules. The analysis revealed statistical significance r (102) = 2.91, p’s < .005.

According to the above investigation, at least on this specific collection of 36 syllogisms, supports the DPTs of multi-propositions that logicians search for counter-examples in MMT. It has extremely higher rates to support the fallacy revealed in this experimental study and somewhere else in the works, however, make it evident from the data as a whole that any such facility must be inadequate. Though it seems that some people may be looking for counterexamples, which effectively leads to the rejection of some of the endorsement fallacies, the lower acceptance rate of PC/ PI rather than NC propositions under □ group rules may be the cause. Logicians need to take into account a different explanation for the lower acceptance percentage of the potential inferences, although individuals will accept any inference that is supported by the first model of the propositions, they consider to be true. Assume the PC/ PI proposition are continually pointed toward a single-model premises which either accept or reject the inference.

As the results, certain PC/ PI propositions would be accepted as happening frequently as NC propositions and others as happening seldom as IPI proposition. This was true for the instantaneous IIT in Experiment I, as they have already mentioned.

A similar tendency was found when the data from the Experiment II IIT under the □ group rules were examined for PC/ PI proposition. Some fallacies were firmly endorsed, such as;

All △ ◁

Some ◁ not ▷

Therefore, some △ not ▷

Here, △ denoted A, ◁ denoted B, and ▷ denoted C. 90% percent students believed the above investigation to be valid under the rules of □ group. Additionally, 90% of the judgements were for **◊** group rules, indicating that (1) individuals have no trouble imagining situations (MM) in which premises and inferences are true but (2) they do not look for examples to prove □ group rules. Considering the classical truth logic that goes like this:

Some △ ◁

All ▷ ◁

Therefore, all ▷ △

When given rules for □ group, just 5% of individuals supported this syllogism; nevertheless, 48% did so when given for **◊** group rules. This implies that individuals first reject the inference as unneeded because they cannot imagine a MM of the premises that would support it in this particular inferences. When individuals are asked if the inference is PC/ PI, it seems as though some effort is made to discover a competing and illustrative MM. The connection between groups MLT rules and logical classification of the propositions stated above might be explained by a tendency to look for other MMs when showing ◊ group rather than □ group. Remember that this collaboration replicates the fact that the rise in inference accepting under the ◊ group rules was greater for PC/PI syllogizes than either NC or IPI.

So, experiment III was developed to both examine the research behind this phenomenon and to validate the correctness of the hypothesizes that few incompatibilities are persistently distributed while others are suppressed.

### Experiment III

As was already said, Experiments I and II have shown that the priori inferences they made regarding the implications of the MMs account are both PC/PI and NC inference. There is another finding but that does not quite fit inside this paradigm. It seems that when PC/PI propositions are evaluated according to □ group rules, some are accepted at extremely high rates corresponding to those of NC propositions, while others are accepted at very low rates corresponding to those connected with IPI difficulties (see above examples of two types of classical truth logic).

One view is that individuals just answer "yes" if the first MM provided by the premises includes the inference, rather than looking for MMs that provide counter-examples. To their knowledge, Experiment II is the only research that has looked at students’ evaluations of all *possible* inferences with all *possible* premises’ types, and it is undoubtedly the first to have done so with assessments of both □ and ◊ group. Although having access to such a database should be beneficial to theorists of all stripes, such a thorough approach has the drawback of requiring each student to complete a very large multi-proposition which can be seen S2 Appendix. The alternate would have essential processed of two hundred fifty-six different computer displays for all students, even maintaining inference rules as between students’ variable. This compelled the separate computer system in which 64 premises pairings were given with four inferences to be assessed.

Similar to the IIT propositions of Experiment I, Experiment III includes presenting chosen modal syllogisms as all student receives only sixty-four propositions overall, each of which is given on 55 separate desktops. Their primary concern in choosing classical truth in syllogistic logic was the notion that there are two types of PC/PI propositions sub-groups i.e., possible consistency (PC) and possible incompatibility (PI) that define as:

**Possible consistency (PC):** Assertions whose inferences are PC but not NC given their propositions, but that frequently endorse as having NC inferences.

**Possible incompatibility (PI):** Assertions whose inferences are PI then not NC assumed their propositions, but that rarely endorse as concerning NC inferences.

In other words, PI syllogistic logic is the fallacy individuals try to avoid whereas, PC syllogistic logic is the fallacy individuals tend to produce (under □ group rules). In the two tests that have been published so far, the identification of these two groups was required to be retroactive. As a result, they made the decision to choose Experiment II’s case of PC/PI propositions with high and low endorsing rates and subject them independently repeat in Experiment III. Table 8 displays the exact syllogistic logic that were chosen. It should be noted that the 64 propositions offered to the 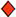

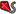
 groups were not always the identical propositions presented to the 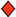

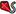
 groups. This is due to the fact that multi-propositions were chosen entirely based on students’ performance how well they performed in Experiment II when given □ group rules.

**Table 8:** On the base of inference rate, classical syllogistic truth logic (CSTL) in Experiment II further investigated in Experiment III.

	M—P P—Q		P—M Q—P		M—P Q—P		P—M P—Q	
CSAL	M Q	Q M	M Q	Q M	M Q	Q M	M Q	Q M
**NC**	∀∀∀	∀~∀~∀	∀∃ ∃	∀∀ ∀	∀~∀~∀	∀~∀~∀	∀∃ ∃	∀∀ ∀
	∀~∀~∀	∃∀ ∃	∀~∀~∃	∀v ∃	∃~∀~∃	∀~∃~∃	∀~∀~∃	∀∃ ∃
	∃∀ ∃	~∀∀~∃	∃~∀~∃	~∀∀~∀	~∀∀~∀	~∀∀~∀	∃~∀~∃	~∀∀~∀
	∃~∀~∃	~∀∃~∃	~∀∀~∀	~∀∃~∃	~∃∀~∃	~∀∃~∃	~∀∀~∀	~∀∃~∃
**IPI**	∀~∀ ∀	∀∀~∀	∀∀~∀	∀∀~∀	∀~∀ ∀	∀~∀ ∀	∀∀~∀	∀∀~∀
**F**	∀~∀ ∃	∀~∀ ∀	∀∃~∀	∀∀~∃	∀~∀ ∃	∀~∃ ∀	∀∃~∀	∀∀~∃
	∃∀~∀	∀~∀ ∃	∃~∀ ∀	~∀∀ ∀	~∀∀ ∀	~∀∀ ∀	∃~∀ ∀	~∀∀ ∀
	∃~∀ ∀	∃∀~∀	~∀∀ ∀	~∀∃ ∀	~∃∀ ∀	~∀∃ ∀	~∀∀ ∀	~∀∃ ∀
**PC**	∀~∃~∃	∀∃ ∃	∀~∃~∃	∃∃ ∃	∀∃~∃	∀∃ ∃	∀~∃~∃∃	∃∃ ∃
	∃∃∃	∃~∃~∃	∃∀ ∃	∃~∃~∃	∃∀ ∃	∃∀ ∃	∃∀ ∃	∃~∃~∃
	~∃∀~∃	~∃∀~∃	∃∃~∃	~∃∀~∃	∃~∃~∃	∃~∃~∃	∃∃~∃	~∃∀~∃
	~∃~∃~∃	~∃∃~∃	~∃∀~∃	~∃∃~∃	~∃∃~∃	~∃∀~∃	~∃∀~∃	~∃∃~∃
**PI**	∃∃~∀	∀~∃ ∀	∀∃ ∀	∀~∃ ∀	∃∀ ∀	∃∀ ∀	∀∃ ∀	∀~∃ ∀
	~∃∃~∀	∃∃ ∀	∀~∃ ∀	∃~∀ ∀	∃∀~∀	∃~∀ ∀	∀~∃ ∀	∃~∀ ∀
	~∃~∀ ∀	∃~∀ ∀	~∃∀ ∀	~∀~∃ ∀	∃∃ ∀	~∀~∃∀	~∃∀ ∀	~∀~∃∀
	~∃~∃ ∀	~∃~∃ ∀	~∃~∃ ∀	~∃~∃ ∀	~∃~∀∀	~∃∃ ∀	~∃~∃∀	~∃~∃∀

**Note**: bold letters denoted propositions pair and small letters denoted inference. As these three types represent, **∀ =** All M P; ~∀ = No M P; ∃ = Some M are P; and ~∃ = Some M not P. M denotes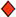, P denotes **▱,** and Q denotes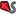.

The classical truth logic that was deemed NC for each four shapes of propositions and inferences order were those that were most commonly endorsed. PC classical truth logic was established by selecting the four greatest frequent endorsed PC/PI propositions in each shape. Similarly, PI propositions were the smallest frequent endorsed. To conclude, the smallest frequent endorsed IPI propositions were designated. This methodology provides a significant experiment of whether PC proposition is endorsed as often as NC proposition, and PI propositions as rarely as IPI propositions. The levels were similar in both situations when given □ group rules that shown in Fig 3.

**Fig 5:**
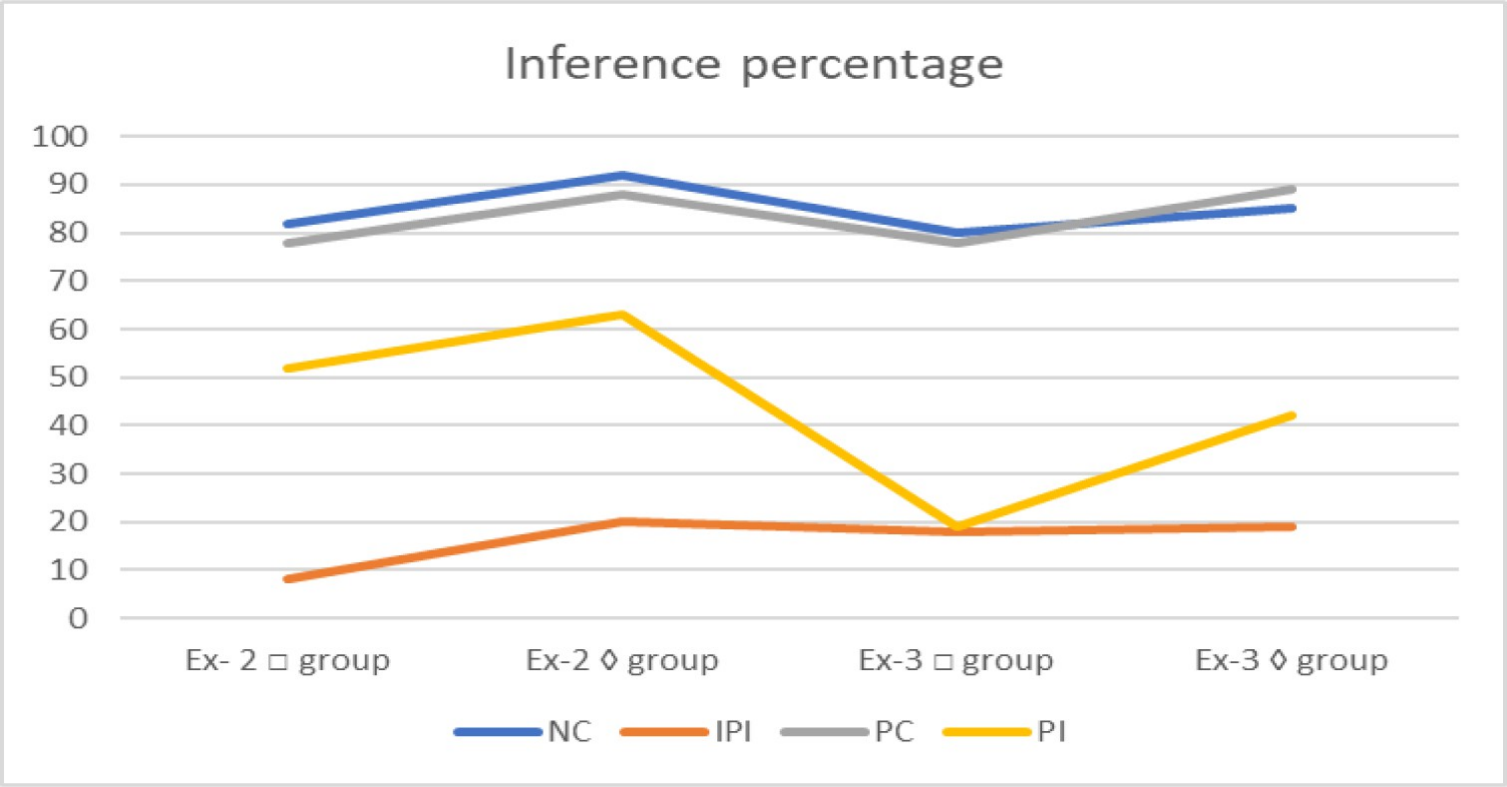
Inferences percentage of Experiment II, □ and ◊ groups rules with four propositions types: NC, PC, PI and IPI, together replicated data from Experiment III.

However, when the identical multi-propositions were considered using **◊** group rules, PI propositions had a somewhat greater acceptance rate than IPI propositions that can be seen in Fig 5. Students in Experiment III got the identical syllogisms whether they responded to **◊** or □ group rules, despite the fact that the □ group results of Experiment II were utilized as the foundation for propositions’ selection.

### Experimental method

#### Students

We determined classical sample size. This experiment was Between-Group design (see Table 9). 40 university students (25 men. 15 women, age average: range 23–30 years) from one university of Pakistan have taken part in this experiment. All are Urdu speakers but their study medium is in English. All of them did not take part in Experiment 1 and 2 or in other similar experiments. To ensure adherence to ethical principles, we got official written approval from the Ethics Committee of Shaanxi Normal University, School of Psychology. Informed written consent was obtained from all study participants, who claimed their understanding of the study’s purposes, and the respondents were assured that their identities and responses were kept confidential which would be maintained in the best interests of academic integrity and every possible manner. Additionally, they were informed of the importance of the study, its possible implications, their role as interviewees, and that no compensation would be offered and they would like to participate in the study voluntarily. Respondents were assured that they could stop responding and withdraw from the interview at any time. The data collected will be used only to suggest mechanisms for abating the impostor’s feelings through academic outcomes.

**Table 9:** Between-subjects factors in Experiment III.

		Value Label	N
Shape	1	Shape A	8
	2	Shape B	8
	3	Shape C	8
	4	Shape D	8
Rules/Inferences	1	**□ -**M P group	16
	2	◊ -Q M group	16
Multi-Propositions	1	NC	8
	2	PC	8
	3	PI	8
	4	IPI	8

M denotes 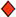, and Q denotes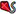.

#### Design

Students 10 each divided in Four groups same as experiment II performed with same rules and inference order □ -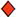

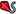
group and ◊ -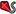

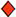
group.

64 separate classical truth logics were given to each student to develop two within student factors, the types of propositions (NC, IPI, PC, and PI), the syllogistic shapes, and repeatedly displayed to each student on four levels each.

#### Materials and procedure

The end and middle phrases utilized in the previously chosen syllogisms, which are displayed in Table 8, were randomly assigned shapes (exclude ∃, and ~∃). Similar to Experiment I, the desktop layout that was employed included two premises and a single inference that could be assessed by clicking YES/NO box. All student saw the 64 multi-propositions on a different desktop, in a randomly chosen order.

The students were first given a general description of the experiment’s design, as well as information about confidentiality and their freedom to withdraw. Depending on the experimental condition they had been given, students were then shown on-desktop rules. With the exception of the *necessity* to assess a particular inference on each desktop, the rules were quite identical to those in Experiment II. Students were given experience using the mouse before the multi-propositions were randomly presented so they could become accustomed to the way Yes and No replies were signaled.

#### Results & discussion

Fig 5 displays the data from Experiment II together with the rates of accepting various logical kinds underneath □ group rules. It is clear that Experiment III offers a tight replication, eliminating the notion that the distinction between PC and PI difficulties was accentuated by a retroactive selection of these types (i.e., due to statistical regression). The acceptance percentages of NC and PC propositions are still quite close in Experiment III.

Only somewhat more frequently than IPI propositions are PI propositions accepted. Again, comparing the results from Experiments II and III, Fig 5 shows accepting rates for the various task types underneath ◊ group rules. A good replication is observed once more, but this time it is more pronounced that PI propositions are more often accepted than IPI propositions. Table 10 provides a breakdown of the mean % acceptance of the conclusions in Experiment III by shapes, multi-propositions, inference/ rules. With four replications of each syllogistic logic, each cell represents the average response of 10 people.

**Table 10:** Inference percentage of Experiment III, divided into propositions type (NC, PC, PI and IPI), Shapes, inference order and both rules’ □ and ◊ groups.

Groups’ Rules &	M—P P—Q		P—M Q—P		M—P Q—P		P—M P—Q		
Propositions type	M Q	Q M	M Q	Q M	M Q	Q M	M Q	Q M	M%
□ Group									
NC	82	78	69	85	77	89	85	78	**80**
IPI	8	23	24	19	14	14	25	12	**18**
PC	85	82	82	73	82	69	72	77	**78**
PI	19	24	23	20	16	14	20	15	**19**
**M%**	**49**	**52**	**50**	**53**	**47**	**47**	**51**	**46**	
◊ group									
NC	92	80	88	87	76	85	88	87	**85**
IPI	32	15	31	7	23	15	29	20	**19**
PC	91	94	88	92	94	91	81	77	**89**
PI	57	25	49	22	47	23	42	26	**42**
**M%**	**68**	**54**	**64**	**52**	**55**	**54**	**60**	**53**	

Here, M denotes 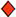, P denotes **▱,** and Q denotes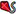.

Logicians were able to conduct 4-way ANOVA on the rate of Yes replies of respondent to the use of replications, using the shapes and Rules as within-group factors and multi-propositions and inference as between-group variables. There were very highly substantial impacts of group rule, F’s (3, 31) = 55.25, SE = 0.89, p’s < .001, and a significant main effect of group rule, F’s (3, 31) = 50.47, SE = 0.134, p’s < .001, where more inferences were accepted under ◊ group (61%) than □ group (39%) rules. Descriptive analysis as under (see Table 11):

**Table 11:** Descriptive statistics of Experiment 3.

	Shapes	Rules/Inferences	Multi-Propositions	Mean	Std. Deviation	N
◊ group	Shapes A	**□—**M P group	NC	62.50	32.522	4
		◊—Q M group	PC	51.75	31.479	4
			Total	57.13	30.182	8
	Shapes B	**□—**M P group	PI	57.25	27.837	4
		◊—Q M group	IPI	53.50	37.546	4
			Total	55.38	30.664	8
	Shapes C	**□—**M P group	NC	54.00	28.902	4
		◊—Q M group	PC	52.75	39.752	4
			Total	53.38	32.182	8
	Shapes D	**□—**M P group	PI	58.75	33.300	4
		◊—Q M group	IPI	51.50	36.263	4
			Total	55.13	32.463	8
	Total	**□—**M P group	NC	58.25	28.843	8
		◊—Q M group	PC	52.25	33.200	8
			Total	52.37	32.555	16
		Total	NC	58.25	28.843	8
			PC	52.25	33.200	8
			PI	58.00	28.425	8
			IPI	52.50	34.189	8
			Total	55.25	29.861	32
□ group	Shapes A	**□—**M P group	NC	54.00	40.538	4
		◊—Q M group	PC	50.00	35.954	4
			Total	52.00	35.537	8
	Shapes B	**□—**M P group	NC	48.75	35.208	4
		◊—Q M group	PC	45.75	36.673	4
			Total	47.25	33.320	8
		Total	NC	51.38	35.262	8
			PC	47.88	33.698	8
			PI	54.88	30.021	8
			IPI	47.75	36.074	8
			Total	50.47	32.302	32

The Multi-Propositions in Experiments I and II (specified on three rather than four types) interacted considerably with the rules, leading to a somewhat higher acceptance of PC/ PI inferences when the rules called for ◊ group. In this analysis, the two variables also strongly interacted, with F’s (3, 182) = 3.75, SE = 0.262, and p’s< .001. However, when the ◊ group is divided into PC and PI, it appears from the means that the primary distinction lies in the accepting rates of PI proposition, which approved rate was 42% underneath ◊ group rules related with just 19% underneath □ group rules. This variance, which is theoretically meaningful and statistically substantial t (55) = 49.365, p’s < .05, demonstrates that when given ◊ group rules, logicians do look for alternate MMs of the propositions if the original one doesn’t support the propositions rather than just answering No. Contrastingly, PC propositions with □ group rules have very high acceptance rates, suggesting that there isn’t much need to look for alternate MMs when the first MM found supports the inference.

There were a number of additional important consequences. First, the main impact of the shapes was significant even if it was simple (F (3, 182) = 3.75, SE = 0.262, and p< .05. The percentage of inferences were accepted ranged from 68% in Fig 1 to 53% in shape 4. Inference order had no major effects, but it did interact with rule in a significant way (F (l, 36) = 7.41, SE = 0.134, p< .001). Looking at Table 12 reveals that this was caused by the ◊ group increased acceptance of the 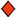

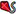
 inferences. This tendency has no readily apparent cause.

**Table 12:** ANOVA of between groups and within groups in Experiment III.

		Sum of Squares	df	Mean Square	F	Sig.
□ group * Shapes	Between Groups	56.500	3	18.833	.019	.996
	Within Groups	27585.500	28	985.196		
	Total	27642.000	31			
◊ group * Shapes	Between Groups	113.094	3	37.698	.033	.992
	Within Groups	32232.875	28	1151.174		
	Total	32345.969	31			
□ group * Rules/Inferences	Between Groups	264.500	1	264.500	.290	.594
	Within Groups	27377.500	30	912.583		
	Total	27642.000	31			
◊ group * Rules/Inferences	Between Groups	225.781	1	225.781	.211	.649
	Within Groups	32120.187	30	1070.673		
	Total	32345.969	31			
□ group * multi-Propositions	Between Groups	265.000	3	88.333	.090	.965
	Within Groups	27377.000	28	977.750		
	Total	27642.000	31			
◊ group * multi-Propositions	Between Groups	274.844	3	91.615	.080	.970
	Within Groups	32071.125	28	1145.397		
	Total	32345.969	31			

The remaining trends included multi-propositions interactions. This was a significant but weak interaction with the shapes and inference separately, as well as with all three types combined. F’s (9, 614) = 1.97, SE = 1.85, p’s < .05. The high repetition of identical results for the similar classical syllogistic logic provided in Experiment III when related with Experiment II challenges some probable criticism of the previous experiment’s design, it should be emphasized. Firstly, participation in Experiment 1 could have had some bearing on how students performed in Experiment II, but Experiment III lacked this prior task. Second, it may be claimed that the enormous number of classical syllogisms utilized in Experiment II made students tired or bored, which could introduce noise into their data. Given the self-pacing that was offered and the fact that the research revealed highly distinct and organized tendencies, they believe that this was unlikely to be accurate in any case. This opinion is supported by the fact that the statistics on equivalent classical syllogisms in Experiment III, when just a fourth as many were utilized, were so similar. There were a number of additional important consequences. First, the main impact of the shapes was significant even if it was simple (F (3, 182) = 3.75, SE = 0.262, and p< .005. The percentage of inferences were accepted ranged from 68% in shape 1 to 53% in shape 4. Inference order had no major effects, but it did interact with rule in a significant way (F (l, 36) = 7.41, SE = 0.134, p< .001). Table 10 reveals that this is caused by the ◊ group increased acceptance of the
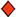

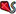
 inferences. This tendency has no readily apparent cause.

## General discussion

This experimental study evaluated the theory that individuals judge whether multi-propositions are necessarily consistent? Individual judgement can decide the validity of inference as either necessarily consistent, possibly consistent or impossibly incompatible. The results showed that logically untrained students are capable of modal inferences on single syllogistic propositions and truth premises. They were also confirmed our main hypothesizes constructed by DPTs.

HP1; Individuals were more likely to endorse inferences as PC/PI rather than NC. This hypothesis suggests an expectation that students are more likely to support an inference that is possible, rather than one that is necessary. This hypothesis implies a tendency for individuals to lean towards more likely or probable conclusions rather than those that are strictly required. According to Experiment 1, in both groups, we computed that the Average Mean percentage of PI/PC (μ = 65.08, SD = 30.095, SE = 8.688, P < .5 and μ = 68.58, SD = 21.047, SE = 6.076, P < .5) exceeds that of NC (μ = 56.50, SD = 16.453, SE = 6.717, P < .5 and μ = 60, SD = 14.895, SE = 6.081, P < .5). This strong confirmation of our hypothesis. Moreover, we conducted A 2x3 ANOVA and subsequent post hoc tests (PHT) to assess the impact of between-subject groups (□ group and ◊ group) and within-subject inference sets (NC vs PC/PI vs IPI). Our analysis focused on the overall mean percentages of inferences endorsed by all students. Each proposition was factored in, utilizing the information provided in three sets for the ANOVA as shown in Table 4. All the results consistently indicate that the ◊ group rules significantly supported students’ judgments, suggesting a possibility of truth. This is evident in the significant rate of .29 for the ◊ group, in contrast to the □ group where the significant rate is zero. This interaction also reflected the high difference among two groups for inference that have possibility. ◊ Group had also a very consequential effect as considering greater recognition under the ◊ group F (l, 27) = 55.96, SE = 5.647, p < .05. This interaction seemed to reflect a larger difference in the inferences that could be drawn between the two groups. Previously, Lee and Wagenmakers [36] used the term possible to compute the possibility of Bayesian analyses. He said that “The Bayesian equivalent of hypothesis testing is model comparison, where competing accounts (possibly, but not necessarily, including a null account) are compared using Bayes factors.” The results of their studies suggested that “logically valid Bayesian analyses are often not possible.” However, we compared inference of possible and necessary in the context of students’ endorsement. Halbach and Welch [37] explained that “There are necessary a posteriori propositions’ or ‘All laws of physics are necessary’ in first-order logic.” He also emphasized as, “Treating truth as a predicate, while necessity and possibly other intentional notions are conceived as operators only. “However, our main focus was on logical truth of modalities, specifically those that are logically consistent and incompatible. However, [38] explained that “In Modal Logic as Metaphysics, Timothy Williamson develops a case for necessities, the theory that necessarily everything necessarily exists”. His main focus was what is the case of necessary. So, most often researches were based on what is necessary case and neglect what is possible case. Thus, our HP1 is contributing significantly for future work directions.

HP2; It’s easier to calculate that inference has PC/ PI if it has also NC. Generally, logicians predict more endorsing PC for NC than for PI propositions.

In Necessity (□) group, the inferences hold in all premise MMs, so premises MMs that individual constructs will support the inference as NC. On the other hands, in ◊ group if the premises support only one PC inference, the individual should look for MM of the premises for which the inferences hold, and ignore those that are contrary to the inferences PI. We observed in experiment 1 that the interaction seemed to reflect a larger difference in the inferences that could be drawn between the two groups. This is not surprising, since the correct answer to these inferences is different among both groups: “Yes” for the ◊ group and “No” for the □ group. The basis for this prediction is that students only need to find one MM that validates the inferences to consider it as PC/PI. In necessary propositions, each MM of the premises supports the inference, while in PC/PI propositions, there is at least one MM that doesn’t. Consequently, it’s expected to be simpler to discover a supporting MM in NC proposition. To test this prediction, one-tailed related-groups t-test was conducted, comparing the endorsement of PC/PI and NC Propositions for the group provided with ◊ group rules. This prediction was significantly agreed, t (39) = 5.44, p < .001. So, we strongly accept our HP2 that single-model rate higher in ◊ group. However, under the ◊ group rules, such tendencies shouldn’t be discovered since in this situation, it’s simply NC to find a model that supports the inferences of the students to prove the answer yes. The patterns in their data support the hypothesis. 18 of the 36 propositions fall into the categories of single-models and multi-models, respectively, according to Johnson-Laird and Bara. When given □ group rules, 82% of propositions with a single-model had their results accepted as opposed to 69% of propositions with multi-models. This tendency was less pronounced under ◊ group rules, with 89% of single-model of multi-propositions accepted vs 78% of multi-model of multi-propositions, as was to be expected. The experiments defined few significant novel evidences for its assumptions. First, Experiment II indicated that the consequence emerged when students were ruled to judge the □ group of the inference—correspondent to the valid inferences performed in traditional reasoning experimental studies of ML theory [39]. Second, the effect was indeed reduced for those students whom were ruled to judge the ◊ group propositions of the inference. This contact significantly suggests that students appreciate that □ group propositions require finding inferences in all MMs, whereas propositions of ◊ group do not. However, this further assumes that students are aware that multi-models of multi-propositions exist but are not sure they have the ability to check all of them due to the constraint of working memory capacity (WMC) [40, 41]. Previously, Polk and Newell [35] claimed different premises interpretations construct external MM drawn through inferences. Students inclined not to recode the premises of single-model syllogisms (as opposed to verbal reasoning). However, they generated a range of MMs for multi-model syllogisms. Either they are looking for counter-examples is less clear or not. However, another occurrence of classical syllogistic logical reasoning supports the examiner for counter-examples. Dietz and Kakas [42] defined selection task contained cognitive reasoning that is appropriately flexible to consistently capture the modifications among the selection of students. Zheng, et al. [43] tested visual reasoning that achieved deep learning and improve accuracy of inference. Image features improve human reasoning models and effective for problem solving. So, that this study used shaped instead of letters for improve the memory capacity and intelligence of university students. As judge counter-examples predicted that students often misrecognized inferences maintained by the preliminary MM of the premise, when in fact they correctly answered that the premise had no inferences. This result suggested that they briefly considered the wrong inference only to reject it because of the counter-example. Moreover, PC/PI propositions were recognized almost same frequently than NC and IPI propositions in both rules in groups, the data under the propositions of □ group appeared to serve multi-modal distribution. 12 PC/ PI propositions, accepted percentage was 48%, same as 10 IPI propositions percentage was 48 and of the 6 NC percentage of inference was 39%. whereas, in ◊ group, PC/ PI inference percentage 61%, NC percentage 44% and percentage of IPI propositions were 55% (see Fig 3) Thus, the results were shown that logicians often did not look for counter-examples, indeed when ruled to develop the □ group findings.

Lastly, HP 3; It’s easier to calculate that inference is not NC if it is also not PC. Generally, logicians predict more PI than IPI proposition endorses as NC. PI inferences hold in at least one MM of the premises, and IPI inferences not at all holds in any one MM of the premises, so that if the individual focuses on a MM that holds, then in the latter case, individual tends to err on the side of inferences. In other words, individuals must find counter-examples to the content of their inferences to draw unnecessary inferences. The search is easier in the case where all MMs of the premises are counter-examples than in the case where at least one is not. These hypothesizes were confirmed in experiment I, in which individuals assessed IIT from an only truth premise to a measured inference, and in experiment II, where we assessed multi-propositions with all *possible* MMs inferences of a syllogistic premise, in experiment III, they assessed sub-groups of MMs inferences drawn from truth logic premises.

As we observed in Experiments that the student’s endorsement of inference more than NC as compare with PC in ◊ group. The data are compatible with the hypothesis that few PC/ PI propositions propose an initial MM that endorses the inferences—inducing to the endorsement of fallacies—while rest one do not. Logicians back to this proposition when they discuss the consequences of Experiment II and provide the reason for Experiment III, all of which involved modal syllogism. Moreover, ML theory rules and classical logical truth classifications significantly reported into Experiment I and II. Examination of Figs 3 and 5 shows that this is due to the difference in endorsement rates between ◊ group and □ group rules being most pronounced on PC/PI syllogisms logic (potential fallacies), which are not sub-groups at first two experiments.

Chen, et al. [44] used rule-based methodology for inference and knowledge graphs method which helps all possible triplets to solve easily. Byrne [45] indicated that MM of the counterfactual inference illusory alternate to truth. Moreover, a MM of the assumed factual truth. Use two method tracking methodology to address such questions. The first approach involved asking students to identify any new inferences they had measured right after their replies, while the second methodology involved creating a circle on a Euler diagram. although strategy shows that students take other models into consideration. Research by Evans, J. S. B., (2019), developed a default-interventionist model combining M and P. This is in line with the data that generally supports intuitive thinking by default, however interventions are also possible.

Initially, one unexpected result was that problems supporting PC/PI inferences fell into two types PC and PI. PC are often considered NC inferences, while PI are rarely considered NC inferences, and sometimes not even considered PI inferences as IPI inferences. This fact was first noticed in experiment I. It also appeared in experiment II, and experiment III, which we planned to contrast two groups of inferences, which we refer to as PC and PI, confirmed it. Of particular here mention in Fig 4 is that PC syllogisms have often recognized as NC, whereas PC and PI syllogisms have rarely recognized as IPI. As we know that this experimental study has lacking counter-example MMs will discuss later. Mostly students have given the responses based on first MM they encountered. As we observed that fallacy is clearly separated into two types, since few sets of premises constantly present a primary model that supports the inferences (PC), while others constantly present a MM that denies the inferences (PI). It is obvious that these results could not be explained by our other hypotheses at a very broad level. Although, the computer program execution of the MMT does generate MMs of explicit sequences, in an effort to support our intuition account, we were able to recognize our two sorts of syllogisms from the program’s output by looking at it.

Results of experiment III have been provided strong confirmation. All the premises studied under the two PC/PI propositions of experiment III are carried out by the program. 29 of the 32 PC syllogisms had an initial MM supporting a given inference, while none of the 32 PI syllogisms had an initial MM supporting a given inference. It observed rise, considering an example of a PC syllogism whose false inference is agreed by a majority of the students:

Some **
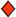
** not ▱

All ▱ 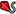


So, some 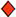 not **
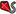
**

Preliminary premises MM are determined by the construct program,

**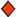 ~**▱

**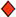 ~**▱

    [▱] **
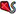
**

    [▱] **
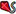
**

Here, ~ denoted negation, **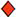,** ▱ and **
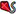
** denoted M, P and Q and braces round ▱ are the later MM, indicates which is fully signified ▱ with reverence to 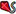. As ▱ could not find in MMs that cannot include 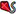. The MM supports the inferences presented, with 78% of students in Experiment III (incorrectly) inferring this to be a PC inference in □ group, and 89% of students (correctly) inferring it to be a PC inference in ◊ group. however, as the software clearly demonstrates, there are other MMs that refute the inferences’ underlying assumptions:

**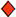 ~**▱ **
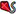
**

**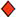 ~**▱ **
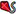
**

    [▱] **
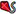
**

    [▱] **
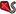
**

So, the inference is PC but not NC. the example of PI classical syllogistic logic has the subsequent way:

All **
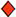
** not ▱

Some 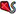 ▱

So, all 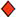 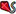
The preliminary MM of premises 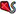 is:

[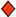] ▱ **
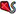
**

[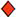] ▱

    
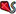


The stated inference is not supported by this MM, but rather the inference "some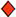 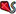 " (or its inverse). Nevertheless, other MMs of the premises can potentially challenge this inference:

[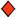] ▱ **
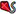
**

    ▱ **
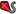
**

[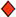] ▱

    ▱ **
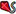
**

To understand that given inferences is at least PI, MLT following syllogism individuals must construct another disjunction of the premises:

[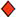] ▱ **
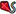
**

[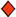] ▱ **
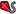
**

    
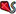


In the combination of premises and inference implies not
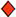
or ▱ but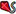. As hypothesized for this interpretation, some students in experiment III incorrectly suggested that a certain inference was □ group (PI = 19%), and only some suggested that it was uniformed ◊ group (PI = 42%). It’s very necessary to know that the finding of this PI syllogism in this experiment is due to the practice involvement, in which students were enquired evaluating all PC/PI inference for all PC/PI pair of premises. In PT approach that guides individuals to a single NC inference, the paired premises in multi-propositions will often link with the generation of few alternative inferences, observed in the example above. A stimulating *possibility* for upcoming research is to ask individuals to draw inferences that are PC rather than NC. Programs that implement MMT work by creating distinct MMs of the two premises and linked them collectively through symbols representing the medium. Consequently, further steps are essential added more symbols to the MM (as we observed above to PC syllogism) or to divided separately (for example [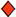] ▱ 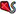) into two separate entities: [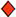] ▱ and ▱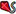 (as we observed to PI inference above). These steps result in a MM built after the preliminary MM. However, our analysis is very encouraging for DPTs, we must be careful that alternative explanations for the difference between PC and PI syllogisms can be offered.

In particular, Reifler, et al. [46] probabilistic heuristic model can account for differences in "minimal heuristics". This postulates that the inference dawn from the syllogistic logical premises that would link the least informative premise of the form, where the informativeness is in the following order: ∀ > ∃ > ~∀ > ~∃. 32 PC syllogisms were shown in Table 2. 30 of inferences matched with the less informative premises, while the remaining 2 were inferences that were least enlightening than either of the premises. For all but one of the 32 PI propositions, the inference was more informative than at least one of the premises. Both the MM procedure and the minimal heuristic provided the difference between PC and PI, and this equally robust explanation in both amazing and motivating. The minimal heuristic is the portion of a completely non-deductive version of classical syllogistic logically reasoning. This has the consequence of ensuring that the argument is not supported when its inference is additional revealing than the premises. This ability holds not individually for all validity propositions, but also for many fallacies. Thus, this theory describes for the fact that students in the classical truth logic experiments endorsed the most valid multi-propositions, but also endorsed many fallacies. Clearly, the modeling procedure is created on a very diverse approach, with the influence of initial producing MMs whose preliminary inferences match the minimal heuristics.

Previously, Grewe [47] discussed such coincidences in terms of "atmospheric effects"—predictions similar to the minimal heuristic. These philosophers also studied the instruction in which the MMT procedure generated the model and observed that, as we do here, errors are often caused by false inferences endorsed by the preliminary MM of the considered premises. Let’s return to the second theory whether individuals judgment is correct, as the last stage of MMT assumes. This question is theoretically important because Polk and Newell [35] place far less emphasis on this stage in their theory of verbal reasoning than in the MMT. This is also an aspect of MMT needed to explain deduction competency.

We noted above were the suggestions for such searches that is the limitation of this experimental study highlighted previously. The fact that other features of our data and data from other research in the literature indicate that at least alternative theories are being explored does not mean we are denying its existence. First, every investigation in the literature, even those carried out by detractors of MMT, indicates that single-model propositions are in fact easier than multi-model propositions. This is a solid conclusion in normal classical syllogistic logic [48]. This distinction may be seen in valid syllogisms where the conclusion is supported by any MMT of the premises. Hence, there must be no modification if people make their decisions just on the first MM.

This experimental study defined few significant novel evidence for its assumptions. First, Experiment II indicated that the consequence emerged when students were ruled to judge the □ group of the inference—correspondent to the valid inferences performed in traditional reasoning experimental studies of MLT [39]. Second, the effect was indeed reduced for those students whom were ruled to judge the ◊ group propositions of the inference. This contact significantly suggests that individuals appreciate that □ group propositions require finding inferences in all MMs, whereas propositions of ◊ group do not. However, this further assumes that people are aware that multi-models of multi-propositions exist but are not sure they have the ability to check all of them due to the constraint of working memory capacity (WMC) [40, 41].

MLT rules and classical truth logic classifications significantly reported into Experiment I and II. Examination of Figs 2 and 3 shows that this is due to the difference in endorsement rates between ◊ group and □ group rules being most pronounced on PC/PI syllogisms logic (potential fallacies), which are not sub-groups at first two experiments. Although the common progress in accepting under the ◊ rule might be due to biases answers (e.g., caution effects), the interaction suggests that at least some individuals search and find alternative MMs. However, an analysis of Experiment III shows that this modification is mainly because of rather significant rise in the accepting rate of the PI syllogism under the ◊ rule. So, we clearly suggested that individuals judge alternative MMs to justify the PC/PI of inferences not considered by the first MM, but in this case, unclear suggestion are that individuals create counter-examples to build the first MM as NC to support inferences.

The question is whether individuals judge multi-propositions of alternative models. However, we can search alternative models reported elsewhere, inferences depending on the methods used different methodologies. Chen, et al. [44] used rule-based methodology for inference and knowledge graphs method which helps all possible triplets to solve easily. Byrne [45] indicated that MM of the counter-factual inference illusory alternate to truth. Moreover, a MM of the assumed factual truth. use two method tracking methodology to address such questions. The first approach involved asking students to identify any new inferences they had measured right after their replies, while the second methodology involved creating a circle on a Euler diagram. although strategy shows that individuals take other models into consideration. Research by Evans, J. S. B., (2019), developed a default-interventionist model combining M and P. This is in line with the data that generally supports intuitive thinking by default, however interventions are also possible. Zhu, et al. [49] presented the model of noise and probability theory framework help individuals to judge systematic bias and improve accuracy through probability estimation. Polk and Newell [35] claimed different premises interpretations construct external MM drawn through inferences. Students inclined not to recode the premises of single-model syllogisms (as opposed to verbal reasoning). However, they generated a range of MMs for multi-model syllogisms. Either they are looking for counter-examples is less clear or not. However, another occurrence of classical syllogistic logical reasoning supports the examiner for counter-examples. Dietz and Kakas [42] defined selection task contained cognitive reasoning that is appropriately flexible to consistently capture the modifications among the selection of individuals. Zheng, et al. [43] tested visual reasoning that achieved deep learning and improve accuracy of inference. Image features improve human reasoning models and effective for problem solving. So, that this study used shaped instead of letters for improve the memory capacity and intelligence of university students. As judge counter-examples predicted that students often misrecognized inferences maintained by the preliminary MM of the premise, when in fact they correctly answered that the premise had no inferences. This result suggested that they briefly considered the wrong inference only to reject it because of the counter-example.

Moreover, the existence of a dozen theories of syllogistic reasoning is an embarrassment to cognitive science [50]. Very few of them are computer simulations. Simulations of model theory yield surprising predictions about human rationality, such as inferences from cognitive fallacies [51]. We know that two factors seem to strongly influence whether such searches are performed. The first one is belief bias effect (BBE), which can be better described as the cautious effect. While this is often described as people’s tendency to support fallacy whom inferences are believable, it can be extra truthfully defined as the partiality to suppress fallacy once their inferences are not believable. Maximum questions practiced in this works were of what we call likely significant type, where fallacies are generally accepted when the inference is neutral and true. These some studies that involved true believable/ unbelievable inferences testified that endorse true inferences was at minimum high as well as believable inferences, and that trust bias was because of inhibition endorsed unbelievable inferences [52–55]. This finding supports a MM explanation of the fallacies effect [56]. Individuals motivated to find counter-examples to invalidate when putative inferences have unbelievable. The effect of MLT rule is also consistent (NC) with the idea that individuals need some stimulus to perform model search. Additionally, a strong pedagogical accent on logical truth necessarily suggestive to compress fallacies, as well as the testified to support possible incompatibility but not necessary consistency inferences. Uncertainty that usual way of rational is inductive than deductive, at that time deductive ability is most clearly believable in situations where people are clearly motivated to try to reason.

## Conclusion

In conclusion, this study helped us to understand of classical truth logic of multi-propositions. We concluded that each individual has different working memory and intelligence, they judge multi-propositions differently. We have described the DPTs that support syllogistic logic especially MMT in the context of BBE [57, 58]. We concluded that when conclusion unbelievable people are motivated to find counterexamples to refute it. It is also consistent with this view people may need some stimulation to get into MM search is the effect of the rules. Previously, Evans, et al. [59] presented the strong rules emphasis on necessity logic greatly reduces BBE and substantially suppressed the tendencies of possible conclusion. However, our finding results endorse the possible as compare with necessary inferences.

The results supported that if the natural way of reasoning is inductive rather than deduction (As stated by Evans and Over [60]) it makes sense then Deductive ability is most evident in specific situations people are obviously motivated to work hard after deductions. Moreover, Modal syllogistics have shown that students endorsed judgment possible rather than necessary inference, and classical truth logic of alethic modalities with syllogism also support inferences those are frequently endorsed multi-models’ inferences. This study has three research questions. First; Individuals will more likely to endorse inferences as PC/PI rather than NC. Second, it’s easier to calculate that inference has PC/ PI if it has also NC. Generally, logicians predict more endorsing PC for NC than for PI proposition. Last, it’s easier to calculate that inference is not NC if it is also not PC. Generally, logicians predict more PI than IPI proposition endorses as NC. All the questions strongly accepted by the students. We also judged the validity of inference. Shapes were used to enhance the working memory and intelligence of students. We also suggested for future that different theories such as probabilistic heuristic model, knowledge modulation heuristics, symmetry, asymmetry etc. can be used to overcome fallacies of inferences when counter-examples were available and helped multi-models inferences of premises. We also suggested that the believes could help to avoid fallacies.

This experimental study has several limitations that we can need to consider when interpreting the results. Firstly, Generalizability: we conducted experiment in controlled settings with a specific sample of students during the epidemic condition in Pakistan, which may not accurately represent the diversity of the larger students’ population of Pakistan. Therefore, it can be challenging to generalize the findings to broader students’ populations or real-world situations. Secondly, Artificial conditions: In order to control for variables and establish cause-and-effect relationships, this experimental study often created artificial conditions that may not fully reflect real-life situations. Students’ behavior in a computer lab setting may differ from their behavior in natural settings, leading to potential discrepancies in the results. Thirdly, Hawthorne effect: Students in experimental studies may alter their behavior or responses simply because they are aware that they are being observed or are part of an experiment. This can lead to an artificial inflation or suppression of certain behaviors or outcomes, impacting the validity of the findings. Lastly, time and resource constraints: we suffered time-consuming and resource-intensive as we face limitations in terms of available funding, sample size, or access to necessary equipment, which may restrict the scope and scale of the study. However, in future we can further gather data in large scale.

## Supporting information

S1 Appendix Experiment I percentage of inference endorsements. (DOCX)

S2 Appendix Experiment II percentage of inference endorsements under rules of □ and ◊ groups. (DOCX)

## References

[1] Lando G., "Metaphysical modality, without possible worlds," in *Thinking and Calculating*: *Essays in Logic*, *Its History and Its Philosophical Applications in Honour of Massimo Mugnai*: Springer, 2022, pp. 385–408.

[2] Leahy B. P. and Carey S. E., "The acquisition of modal concepts," *Trends in Cognitive Sciences*, vol. 24, no. 1, pp. 65–78, 2020. doi: 10.1016/j.tics.2019.11.004 31870542

[3] Kripke S. A., "Semantical analysis of modal logic i normal modal propositional calculi," *Mathematical Logic Quarterly*, vol. 9, no. 5‐6, pp. 67–96, 1963.

[4] Meytus V. Y., "Problems of constructing intelligent systems. Intelligent modeling," *Cybernetics and Systems Analysis*, vol. 57, pp. 509–520, 2021.

[5] Omnes R., "Logical reformulation of quantum mechanics. I. Foundations," *Journal of Statistical Physics*, vol. 53, pp. 893–932, 1988.

[6] Heck R. G., "On the consistency of second-order contextual definitions," *Noûs*, vol. 26, no. 4, pp. 491–494, 1992.

[7] Zacharopoulou D., Skopeliti A., and Nakos B., "Assessment and Visualization of OSM Consistency for European Cities," *ISPRS International Journal of Geo-Information*, vol. 10, no. 6, p. 361, 2021.

[8] Perach B., Ronnen R., and Kvatinsky S., "On Consistency for Bulk-Bitwise Processing-in-Memory," *arXiv preprint arXiv*:*2211*.*07542*, 2022.

[9] Perrier E., "Computability, Complexity, Consistency and Controllability: A Four C’s Framework for cross-disciplinary Ethical Algorithm Research," *arXiv preprint arXiv*:*2102*.*04234*, 2021.

[10] Bunder M. W., "Consistency notions in illative combinatory logic," *The Journal of Symbolic Logic*, vol. 42, no. 4, pp. 527–529, 1977.

[11] Berto F. and Restall G., "Negation on the Australian plan," *Journal of Philosophical Logic*, vol. 48, no. 6, pp. 1119–1144, 2019.

[12] Peregrin J., "Logic as based on incompatibility," *The logica yearbook*, pp. 157–168, 2010.

[13] Knowles W. B., "Is Colour incompatibility analytic?," *Ratio*, 2023.

[14] Jacquette D., "Tractatus Objects and the Logic of Color Incompatibility," *Colours in the development of Wittgenstein’s Philosophy*, pp. 57–94, 2017.

[15] Thom P., "Logic and metaphysics in Avicenna’s modal syllogistic," *The Unity of Science in the Arabic Tradition*: *Science*, *Logic*, *Epistemology and Their Interactions*, pp. 361–376, 2008.

[16] Smith R., *Prior analytics*. Hackett Publishing, 1989.

[17] Johnson-Laird P. N. and Bara B. G., "Syllogistic inference," *Cognition*, vol. 16, no. 1, pp. 1–61, 1984. doi: 10.1016/0010-0277(84)90035-0 6540648

[18] Bucciarelli M. and Johnson‐Laird P. N., "Strategies in syllogistic reasoning," *Cognitive Science*, vol. 23, no. 3, pp. 247–303, 1999.

[19] Lockwood D., *Confused by the Odds*: *How Probability Misleads Us*. Greenleaf Book Group, 2023.

[20] Rips L. J., *The psychology of proof*: *Deductive reasoning in human thinking*. Mit Press, 1994.

[21] Poucher Z. A., Tamminen K. A., Caron J. G., and Sweet S. N., "Thinking through and designing qualitative research studies: A focused mapping review of 30 years of qualitative research in sport psychology," *International Review of Sport and Exercise Psychology*, vol. 13, no. 1, pp. 163–186, 2020.

[22] Montero I. and León O. G., "A guide for naming research studies in Psychology," *International Journal of clinical and Health psychology*, vol. 7, no. 3, pp. 847–862, 2007.

[23] Noveck I. A., "When children are more logical than adults: Experimental investigations of scalar implicature," *Cognition*, vol. 78, no. 2, pp. 165–188, 2001. doi: 10.1016/s0010-0277(00)00114-1 11074249

[24] Kment B., *Modality and explanatory reasoning*. OUP Oxford, 2014.

[25] Doligez D., Kriener J., Lamport L., Libal T., and Merz S., "Coalescing: Syntactic abstraction for reasoning in first-order modal logics," *arXiv preprint arXiv*:*1409*.*3819*, 2014.

[26] Carreira S., Amado N., and Jacinto H., "Venues for analytical reasoning problems: How children produce deductive reasoning," *Education Sciences*, vol. 10, no. 6, p. 169, 2020.

[27] Schechter J., "Deductive reasoning," 2013.

[28] Johnson-Laird P. N., "Mental models, deductive reasoning, and the brain," *The cognitive neurosciences*, vol. 65, pp. 999–1008, 1995.

[29] Johnson-Laird P. N., "Deductive reasoning," *Annual review of psychology*, vol. 50, no. 1, pp. 109–135, 1999. doi: 10.1146/annurev.psych.50.1.109 15012459

[30] Khemlani S., Lotstein M., Trafton J. G., and Johnson-Laird P. N., "Immediate inferences from quantified assertions," *Quarterly Journal of Experimental Psychology*, vol. 68, no. 10, pp. 2073–2096, 2015. doi: 10.1080/17470218.2015.1007151 25607245

[31] Kantiloros S., "The Role of Context and Semantics in Reasoning: Understanding the Normative/Descriptive Gap," 2020.

[32] Bara B. G., Bucciarelli M., and Johnson-Laird P. N., "Development of syllogistic reasoning," *The American journal of psychology*, pp. 157–193, 1995. 7625491

[33] Gilhooly K., Logie R., Wetherick N., and Wynn V., "Working memory and strategies in syllogistic-reasoning tasks," *Memory & Cognition*, vol. 21, pp. 115–124, 1993. doi: 10.3758/bf03211170 8433642

[34] Endrullis J. and Moss L. S., "Syllogistic logic with “Most”," *Mathematical Structures in Computer Science*, vol. 29, no. 6, pp. 763–782, 2019.

[35] Polk T. A. and Newell A., "Deduction as verbal reasoning," *Psychological Review*, vol. 102, no. 3, p. 533, 1995.

[36] Lee M. D. and Wagenmakers E.-J., "Bayesian statistical inference in psychology: comment on Trafimow (2003)," 2005. 10.1037/0033-295X.112.3.662 16060758

[37] Halbach V. and Welch P., "Necessities and necessary truths: A prolegomenon to the use of modal logic in the analysis of intensional notions," *Mind*, vol. 118, no. 469, pp. 71–100, 2009.

[38] Sullivan M., "Modal logic as methodology," *Philosophy and Phenomenological Research*, vol. 88, no. 3, pp. 734–743, 2014.

[39] Braine M. D. and O’Brien D. P., *Mental logic*. Psychology Press, 1998.

[40] Quelhas A. C., Rasga C., and Johnson‐Laird P. N., "A priori true and false conditionals," *Cognitive Science*, vol. 41, pp. 1003–1030, 2017. doi: 10.1111/cogs.12479 28370159

[41] Johnson-Laird P. N., "Mental models and deduction," *Trends in cognitive sciences*, vol. 5, no. 10, pp. 434–442, 2001. doi: 10.1016/s1364-6613(00)01751-4 11707382

[42] Dietz E. and Kakas A., "Cognitive argumentation and the selection task," in *Proceedings of the annual meeting of the Cognitive Science Society*, 2021, vol. 43, no. 43.

[43] Zheng W., Liu X., Ni X., Yin L., and Yang B., "Improving visual reasoning through semantic representation," *IEEE access*, vol. 9, pp. 91476–91486, 2021.

[44] Chen B., Wu M., Zheng B., Zhu S., and Peng W., "Predicate Logic Network: Vision Concept Formation," in *Artificial Intelligence Logic and Applications*: *The 2nd International Conference*, *AILA 2022*, *Shanghai*, *China*, *August 26–28*, *2022*, *Proceedings*, 2022: Springer, pp. 35–48.

[45] Byrne R. M., "Counterfactuals in Explainable Artificial Intelligence (XAI): Evidence from Human Reasoning," in *IJCAI*, 2019, pp. 6276–6282.

[46] Reifler J., Clarke H. D., Scotto T. J., Sanders D., Stewart M. C., and Whiteley P., "Prudence, principle and minimal heuristics: British public opinion toward the use of military force in Afghanistan and Libya," *The British Journal of Politics and International Relations*, vol. 16, no. 1, pp. 28–55, 2014.

[47] Grewe L. L., "Detecting and counteracting atmospheric effects," *Distributed Sensor Networks*, pp. 213–224, 2004.

[48] Johnson-Laird P. N., "Mental models and cognitive change," *Journal of Cognitive Psychology*, vol. 25, no. 2, pp. 131–138, 2013.

[49] Zhu J.-Q., Sanborn A. N., and Chater N., "The Bayesian sampler: Generic Bayesian inference causes incoherence in human probability judgments," *Psychological review*, vol. 127, no. 5, p. 719, 2020. doi: 10.1037/rev0000190 32191073 PMC7571263

[50] Kelly L. J., Khemlani S., and Johnson-Laird P. N., "Reasoning about durations," *Journal of Cognitive Neuroscience*, vol. 32, no. 11, pp. 2103–2116, 2020. doi: 10.1162/jocn_a_01621 32812828

[51] Khemlani S. and Johnson-Laird P. N., "Reasoning about properties: A computational theory," *Psychological Review*, vol. 129, no. 2, p. 289, 2022. doi: 10.1037/rev0000240 34553967

[52] Over D., "Dual process theory 2.0," ed: Taylor & Francis, 2020.

[53] Andrews G. and Vann D. M., "Solving distant analogies reduces belief-based responding in transitive inference," *Journal of Cognitive Psychology*, vol. 31, no. 7, pp. 760–767, 2019.

[54] Andrews G., "Belief-based and analytic processing in transitive inference depends on premise integration difficulty," *Memory & cognition*, vol. 38, pp. 928–940, 2010. doi: 10.3758/MC.38.7.928 20921105

[55] Evans J. S. B., Handley S. J., and Bacon A. M., "Reasoning under time pressure: A study of causal conditional inference," *Experimental Psychology*, vol. 56, no. 2, pp. 77–83, 2009. 19261582 10.1027/1618-3169.56.2.77

[56] Brisson J., Markovits H., Robert S., and Schaeken W., "Reasoning from an incompatibility: False dilemma fallacies and content effects," *Memory & Cognition*, vol. 46, pp. 657–670, 2018. doi: 10.3758/s13421-018-0804-x 29572787

[57] Torrens D., "Individual differences and the belief bias effect: Mental models, logical necessity, and abstract reasoning," *Thinking & Reasoning*, vol. 5, no. 1, pp. 1–28, 1999.

[58] Newstead S. E., Pollard P., Evans J. S. B., and Allen J. L., "The source of belief bias effects in syllogistic reasoning," *Cognition*, vol. 45, no. 3, pp. 257–284, 1992. doi: 10.1016/0010-0277(92)90019-e 1490324

[59] Evans J. S. B., Newstead S., Allen J., and Pollard P., "Debiasing by instruction: The case of belief bias," *European Journal of Cognitive Psychology*, vol. 6, no. 3, pp. 263–285, 1994.

[60] Evans J. S. B. and Over D. E., "Reasoning to and from belief: Deduction and induction are still distinct," *Thinking & Reasoning*, vol. 19, no. 3–4, pp. 267–283, 2013.

