# Supplementary material for: An experimental study of classical truth logic on multi-propositions consistent and incompatible: Dual-process theories and modal syllogistic of deduction
Source: PLoS One. 2024 Jul 2;19(7):e0299741. doi: 10.1371/journal.pone.0299741 (PMC11218998; doi:10.1371/journal.pone.0299741)
Supplement: S1 Appendix — (DOCX) [file pone.0299741.s001.docx]

**Appendix 1**

Experiment I percentage of inference endorsements.

| **Premises** | **Inferences** | **Alethic Logic** | **□ group** | **◊ group** |
| --- | --- | --- | --- | --- |
| All M are P | Some M are P | NC | 38 | 65 |
|  | No M are P | IPI | 53 | 79 |
|  | Some M are not P | IPI | 8 | 45 |
|  | All P are M | PC/PI | 65 | 78 |
|  | Some P are M | NC | 70 | 35 |
|  | No P are M | IPI | 10 | 38 |
|  | Some P are not M | PC/PI | 76 | 85 |
|  |  |  |  |  |
| Some M are P | All M are P | PC/PI | 65 | 95 |
|  | No M are P | IPI | 10 | 93 |
|  | Some M are not P | PC/PI | 75 | 45 |
|  | All P are M | PC/PI | 18 | 33 |
|  | Some P are M | NC | 58 | 68 |
|  | No P are M | IPI | 18 | 35 |
|  | Some P are not M | PC/PI | 83 | 55 |
|  |  |  |  |  |
| No M are P | All M are P | IPI | 5 | 8 |
|  | Some M are P | IPI | 5 | 9 |
|  | Some M are not P | NC | 78 | 75 |
|  | All P are M | IPI | 10 | 33 |
|  | Some P are M | IPI | 13 | 98 |
|  | No P are M | NC | 50 | 38 |
|  | Some P are not M | NC | 70 | 58 |
|  |  |  |  |  |
| Some M are not P | All M are P | IPI | 4 | 9 |
|  | Some M are P | PC/PI | 90 | 23 |
|  | No M are P | PC/PI | 78 | 93 |
|  | All P are M | PC/PI | 40 | 88 |
|  | Some P are M | PC/PI | 80 | 13 |
|  | No P are M | PC/PI | 63 | 75 |
|  | Some P are not M | PC/PI | 90 | 98 |

Note: here, Diamond (♦️) denotes M and Rhombus (▱) denotes P. necessary consistency (NC), possible consistency (PC) or possible incompatibility (PI) and impossible incompatibility (IPI)
