# Supplementary material for: An experimental study of classical truth logic on multi-propositions consistent and incompatible: Dual-process theories and modal syllogistic of deduction
Source: PLoS One. 2024 Jul 2;19(7):e0299741. doi: 10.1371/journal.pone.0299741 (PMC11218998; doi:10.1371/journal.pone.0299741)
Supplement: S2 Appendix — (DOCX) [file pone.0299741.s002.docx]

**Appendix 2**

Experiment II percentage of inference endorsements under rules of □ and ◊ groups.

| S | **Inf.**  **order** | **propositions** | **inferences** | **AL** | **□** | **◊** | **S** | **Inf.**  **order** | **propositions** | **inferences** | **AL** | **□** | **◊** |
| --- | --- | --- | --- | --- | --- | --- | --- | --- | --- | --- | --- | --- | --- |
| 1 | M - Q | All M are P | All M are Q | NC | 75 | 82 | 1 | M - Q | All M are P | All M are Q | IPI | 5 | 9 |
|  |  | All P are Q | Some M are Q | NC | 25 | 54 |  |  | No P are Q | Some M are Q | IPI | 9 | 17 |
|  |  |  | No M are Q | IPI | 9 | 29 |  |  |  | No M are Q | NC | 81 | 98 |
|  |  |  | Some M are not Q | IPI | 21 | 17 |  |  |  | Some M are not Q | NC | 29 | 58 |
| 1 | Q - M | All M are P | All Q are M | PC/PI | 75 | 84 | 1 | Q - M | All M are P | All Q are M | IPI | 9 | 10 |
|  |  | All P are Q | Some Q are M | NC | 51 | 67 |  |  | No P are Q | Some Q are M | IPI | 9 | 18 |
|  |  |  | No Q are M | IPI | 8 | 21 |  |  |  | No Q are M | NC | 86 | 89 |
|  |  |  | Some Q are not M | PC/PI | 23 | 47 |  |  |  | Some Q are not M | NC | 43 | 53 |
| 1 | M - Q | All M are P | All M are Q | PC/PI | 79 | 75 | 1 | M - Q | All M are P | All M are Q | PC/PI | 9 | 11 |
|  |  | Some P are Q | Some M are Q | PC/PI | 52 | 62 |  |  | Some P are not Q | Some M are Q | PC/PI | 84 | 87 |
|  |  |  | No M are Q | PC/PI | 8 | 24 |  |  |  | No M are Q | PC/PI | 44 | 56 |
|  |  |  | Some M are not Q | PC/PI | 81 | 89 |  |  |  | Some M are not Q | PC/PI | 90 | 91 |
| 1 | Q - M | All M are P | All Q are M | PC/PI | 9 | 34 | 1 | Q - M | All M are P | All Q are M | PC/PI | 7 | 23 |
|  |  | Some P are Q | Some Q are M | PC/PI | 86 | 92 |  |  | Some P are not Q | Some Q are M | PC/PI | 45 | 86 |
|  |  |  | No Q are M | PC/PI | 14 | 27 |  |  |  | No Q are M | PC/PI | 25 | 28 |
|  |  |  | Some Q are not M | PC/PI | 82 | 89 |  |  |  | Some Q are not M | PC/PI | 76 | 93 |
| 1 | M - Q | Some M are P | All M are Q | PC/PI | 9 | 21 | 1 | M - Q | Some M are P | All M are Q | IPI | 0 | 15 |
|  |  | All P are Q | Some M are Q | NC | 88 | 92 |  |  | No P are Q | Some M are Q | PC/PI | 37 | 68 |
|  |  |  | No M are Q | IPI | 13 | 23 |  |  |  | No M are Q | PC/PI | 42 | 79 |
|  |  |  | Some M are not Q | PC/PI | 84 | 87 |  |  |  | Some M are not Q | NC | 87 | 98 |
| 1 | Q - M | Some M are P | All Q are M | PC/PI | 19 | 29 | 1 | Q - M | Some M are P | All Q are M | PC/PI | 4 | 23 |
|  |  | All P are Q | Some Q are M | NC | 87 | 90 |  |  | No P are Q | Some Q are M | PC/PI | 51 | 49 |
|  |  |  | No Q are M | IPI | 9 | 21 |  |  |  | No Q are M | PC/PI | 26 | 27 |
|  |  |  | Some Q are not M | PC/PI | 82 | 86 |  |  |  | Some Q are not M | PC/PI | 77 | 94 |
| 1 | M - Q | Some M are P | All M are Q | PC/PI | 9 | 31 | 1 | M - Q | Some M are P | All M are Q | PC/PI | 4 | 23 |
|  |  | Some P are Q | Some M are Q | PC/PI | 71 | 99 |  |  | Some P are not Q | Some M are Q | PC/PI | 51 | 49 |
|  |  |  | No M are Q | PC/PI | 8 | 51 |  |  |  | No M are Q | PC/PI | 47 | 78 |
|  |  |  | Some M are not Q | PC/PI | 79 | 94 |  |  |  | Some M are not Q | PC/PI | 61 | 71 |
| 1 | Q - M | Some M are P | All Q are M | IPI | 5 | 48 | 1 | Q - M | Some M are P | All Q are M | PC/PI | 15 | 43 |
|  |  | Some P are Q | Some Q are M | PC/PI | 69 | 89 |  |  | Some P are not Q | Some Q are M | PC/PI | 69 | 99 |
|  |  |  | No Q are M | PC/PI | 84 | 92 |  |  |  | No Q are M | PC/PI | 9 | 58 |
|  |  |  | Some Q are not M | NC | 4 | 43 |  |  |  | Some Q are not M | PC/PI | 67 | 95 |
| 1 | M - Q | No M are P | All M are Q | PC/PI | 71 | 84 | 1 | M - Q | No M are P | All M are Q | PC/PI | 9 | 37 |
|  |  | All P are Q | Some M are Q | PC/PI | 4 | 53 |  |  | No P are Q | Some M are Q | PC/PI | 58 | 92 |
|  |  |  | No M are Q | PC/PI | 78 | 91 |  |  |  | No M are Q | PC/PI | 19 | 48 |
|  |  |  | Some M are not Q | PC/PI | 46 | 72 |  |  |  | Some M are not Q | PC/PI | 78 | 100 |
| 1 | Q - M | No M are P | All Q are M | IPI | 16 | 24 | 1 | Q - M | No M are P | All Q are M | PC/PI | 17 | 37 |
|  |  | All P are Q | Some Q are M | PC/PI | 27 | 29 |  |  | No P are Q | Some Q are M | PC/PI | 26 | 42 |
|  |  |  | No Q are M | PC/PI | 56 | 57 |  |  |  | No Q are M | PC/PI | 63 | 72 |
|  |  |  | Some Q are not M | NC | 9 | 23 |  |  |  | Some Q are not M | PC/PI | 45 | 75 |
| 1 | M - Q | No M are P | All M are Q | PC/PI | 67 | 86 | 1 | M - Q | No M are not P | All M are Q | PC/PI | 15 | 32 |
|  |  | Some P are Q | Some M are Q | PC/PI | 18 | 32 |  |  | Some P are not Q | Some M are Q | PC/PI | 31 | 41 |
|  |  |  | No M are Q | PC/PI | 71 | 38 |  |  |  | No M are Q | PC/PI | 54 | 83 |
|  |  |  | Some M are not Q | PC/PI | 83 | 96 |  |  |  | Some M are not Q | PC/PI | 41 | 61 |
| 1 | Q - M | No M are P | All Q are M | PC/PI | 15 | 24 | 1 | Q - M | No M are not P | All Q are M | PC/PI | 12 | 41 |
|  |  | Some P are Q | Some Q are M | PC/PI | 82 | 92 |  |  | Some P are not Q | Some Q are M | PC/PI | 42 | 85 |
|  |  |  | No Q are M | PC/PI | 9 | 41 |  |  |  | No Q are M | PC/PI | 42 | 68 |
|  |  |  | Some Q are not M | PC/PI | 64 | 89 |  |  |  | Some Q are not M | PC/PI | 53 | 78 |
| 1 | M - Q | Some M are not P | All M are Q | PC/PI | 19 | 43 | 1 | M - Q | Some M are not P | All M are Q | PC/PI | 19 | 31 |
|  |  | All P are Q | Some M are Q | PC/PI | 82 | 83 |  |  | No P are Q | Some M are Q | PC/PI | 43 | 71 |
|  |  |  | No M are Q | PC/PI | 17 | 55 |  |  |  | No M are Q | PC/PI | 41 | 68 |
|  |  |  | Some M are not Q | PC/PI | 78 | 81 |  |  |  | Some M are not Q | PC/PI | 64 | 78 |
| 1 | Q - M | Some M are not P | All Q are M | PC/PI | 4 | 43 | 1 | Q - M | Some M are not P | All Q are M | PC/PI | 42 | 47 |
|  |  | All P are Q | Some Q are M | PC/PI | 57 | 85 |  |  | No P are Q | Some Q are M | PC/PI | 57 | 89 |
|  |  |  | No Q are M | PC/PI | 25 | 58 |  |  |  | No Q are M | PC/PI | 19 | 31 |
|  |  |  | Some Q are not M | PC/PI | 86 | 89 |  |  |  | Some Q are not M | PC/PI | 67 | 94 |
| 1 | M - Q | Some M are not P | All M are Q | PC/PI | 19 | 45 | 1 | M - Q | Some M are not P | All M are Q | PC/PI | 9 | 36 |
|  |  | Some P are Q | Some M are Q | PC/PI | 84 | 94 |  |  | Some P are not Q | Some M are Q | PC/PI | 79 | 79 |
|  |  |  | No M are Q | PC/PI | 17 | 32 |  |  |  | No M are Q | PC/PI | 19 | 45 |
|  |  |  | Some M are not Q | PC/PI | 72 | 82 |  |  |  | Some M are not Q | PC/PI | 85 | 95 |
| 1 | Q - M | Some M are not P | All Q are M | PC/PI | 9 | 37 | 1 | Q - M | Some M are not P | All Q are M | PC/PI | 0 | 31 |
|  |  | Some P are Q | Some Q are M | PC/PI | 53 | 86 |  |  | Some P are not Q | Some Q are M | PC/PI | 54 | 89 |
|  |  |  | No Q are M | PC/PI | 26 | 57 |  |  |  | No Q are M | PC/PI | 19 | 34 |
|  |  |  | Some Q are not M | PC/PI | 84 | 84 |  |  |  | Some Q are not M | PC/PI | 65 | 95 |

**Appendix 2 (to be continue)**

| **S** | **Inf.**  **order** | **propositions** | **inferences** | **AL** | **□** | **◊** | **S** | **Inf.**  **order** | **propositions** | **inferences** | **AL** | **□** | **◊** |
| --- | --- | --- | --- | --- | --- | --- | --- | --- | --- | --- | --- | --- | --- |
| 2 | M - Q | All P are M | All M are Q | PC/PI | 74 | 82 | 2 | M - Q | All P are M | All M are Q | IPI | 15 | 19 |
|  |  | All Q are P | Some M are Q | NC | 45 | 54 |  |  | No Q are P | Some M are Q | PC/PI | 19 | 27 |
|  |  |  | No M are Q | IPI | 11 | 29 |  |  |  | No M are Q | PC/PI | 44 | 88 |
|  |  |  | Some M are not Q | PC/PI | 9 | 17 |  |  |  | Some M are not Q | NC | 16 | 58 |
| 2 | Q - M | All P are M | All Q are M | NC | 4 | 84 | 2 | Q - M | All P are M | All Q are M | PC/PI | 11 | 24 |
|  |  | All Q are P | Some Q are M | NC | 88 | 67 |  |  | No Q are P | Some Q are M | PC/PI | 21 | 48 |
|  |  |  | No Q are M | IPI | 8 | 21 |  |  |  | No Q are M | PC/PI | 46 | 82 |
|  |  |  | Some Q are not M | IPI | 76 | 47 |  |  |  | Some Q are not M | PC/PI | 43 | 53 |
| 2 | M - Q | All P are M | All M are Q | PC/PI | 11 | 75 | 2 | M - Q | All P are M | All M are Q | PC/PI | 9 | 11 |
|  |  | Some Q are P | Some M are Q | NC | 85 | 62 |  |  | Some Q are not P | Some M are Q | PC/PI | 84 | 87 |
|  |  |  | No M are Q | IPI | 14 | 24 |  |  |  | No M are Q | PC/PI | 44 | 56 |
|  |  |  | Some M are not Q | PC/PI | 57 | 89 |  |  |  | Some M are not Q | PC/PI | 90 | 91 |
| 2 | Q - M | All P are M | All Q are M | PC/PI | 11 | 34 | 2 | Q - M | All P are M | All Q are M | PC/PI | 7 | 23 |
|  |  | Some Q are P | Some Q are M | PC/PI | 76 | 92 |  |  | Some Q are not P | Some Q are M | PC/PI | 45 | 86 |
|  |  |  | No Q are M | PC/PI | 9 | 27 |  |  |  | No Q are M | PC/PI | 25 | 28 |
|  |  |  | Some Q are not M | PC/PI | 82 | 89 |  |  |  | Some Q are not M | PC/PI | 76 | 93 |
| 2 | M - Q | Some P are M | All M are Q | PC/PI | 3 | 21 | 2 | M - Q | Some P are M | All M are Q | IPI | 9 | 15 |
|  |  | All Q are P | Some M are Q | PC/PI | 78 | 92 |  |  | No Q are P | Some M are Q | PC/PI | 37 | 68 |
|  |  |  | No M are Q | PC/PI | 43 | 23 |  |  |  | No M are Q | PC/PI | 42 | 79 |
|  |  |  | Some M are not Q | PC/PI | 77 | 87 |  |  |  | Some M are not Q | NC | 87 | 98 |
| 2 | Q - M | Some P are M | All Q are M | PC/PI | 4 | 29 | 2 | Q - M | Some P are M | All Q are M | PC/PI | 4 | 23 |
|  |  | All Q are P | Some Q are M | PC/PI | 37 | 90 |  |  | No Q are P | Some Q are M | PC/PI | 51 | 49 |
|  |  |  | No Q are M | PC/PI | 9 | 21 |  |  |  | No Q are M | PC/PI | 56 | 27 |
|  |  |  | Some Q are not M | PC/PI | 82 | 86 |  |  |  | Some Q are not M | PC/PI | 57 | 94 |
| 2 | M - Q | Some P are M | All M are Q | PC/PI | 12 | 31 | 2 | M - Q | Some P are M | All M are Q | PC/PI | 0 | 23 |
|  |  | Some Q are P | Some M are Q | PC/PI | 82 | 109 |  |  | Some Q are not P | Some M are Q | PC/PI | 51 | 49 |
|  |  |  | No M are Q | PC/PI | 8 | 51 |  |  |  | No M are Q | PC/PI | 47 | 78 |
|  |  |  | Some M are not Q | PC/PI | 79 | 94 |  |  |  | Some M are not Q | PC/PI | 61 | 71 |
| 2 | Q - M | Some P are M | All Q are M | PC/PI | 5 | 18 | 2 | Q - M | Some P are M | All Q are M | PC/PI | 15 | 43 |
|  |  | Some Q are P | Some Q are M | PC/PI | 69 | 78 |  |  | Some Q are not P | Some Q are M | PC/PI | 69 | 99 |
|  |  |  | No Q are M | PC/PI | 84 | 92 |  |  |  | No Q are M | PC/PI | 9 | 58 |
|  |  |  | Some Q are not M | PC/PI | 32 | 71 |  |  |  | Some Q are not M | PC/PI | 67 | 95 |
| 2 | M - Q | No P are M | All M are Q | IPI | 9 | 15 | 2 | M - Q | No P are M | All M are Q | PC/PI | 4 | 37 |
|  |  | All Q are P | Some M are Q | IPI | 9 | 18 |  |  | No Q are P | Some M are Q | PC/PI | 58 | 92 |
|  |  |  | No M are Q | NC | 78 | 91 |  |  |  | No M are Q | PC/PI | 9 | 48 |
|  |  |  | Some M are not Q | NC | 46 | 72 |  |  |  | Some M are not Q | PC/PI | 78 | 100 |
| 2 | Q - M | No P are M | All Q are M | IPI | 16 | 24 | 2 | Q - M | No P are M | All Q are M | PC/PI | 17 | 37 |
|  |  | All Q are P | Some Q are M | IPI | 10 | 27 |  |  | No Q are P | Some Q are M | PC/PI | 26 | 42 |
|  |  |  | No Q are M | NC | 56 | 87 |  |  |  | No Q are M | PC/PI | 53 | 72 |
|  |  |  | Some Q are not M | NC | 32 | 62 |  |  |  | Some Q are not M | PC/PI | 45 | 75 |
| 2 | M - Q | No P are M | All M are Q | PC/PI | 67 | 86 | 2 | M - Q | No P are not M | All M are Q | PC/PI | 15 | 32 |
|  |  | Some Q are P | Some M are Q | PC/PI | 18 | 32 |  |  | Some Q are not P | Some M are Q | PC/PI | 31 | 41 |
|  |  |  | No M are Q | PC/PI | 71 | 38 |  |  |  | No M are Q | PC/PI | 54 | 83 |
|  |  |  | Some M are not Q | PC/PI | 83 | 96 |  |  |  | Some M are not Q | PC/PI | 41 | 61 |
| 2 | Q - M | No P are M | All Q are M | IPI | 4 | 20 | 2 | Q - M | No P are not M | All Q are M | PC/PI | 12 | 41 |
|  |  | Some Q are P | Some Q are M | PC/PI | 82 | 92 |  |  | Some Q are not P | Some Q are M | PC/PI | 52 | 85 |
|  |  |  | No Q are M | PC/PI | 9 | 41 |  |  |  | No Q are M | PC/PI | 32 | 68 |
|  |  |  | Some Q are not M | NC | 64 | 89 |  |  |  | Some Q are not M | PC/PI | 53 | 78 |
| 2 | M - Q | Some P are not M | All M are Q | PC/PI | 19 | 43 | 2 | M - Q | Some P are not M | All M are Q | PC/PI | 0 | 31 |
|  |  | All Q are P | Some M are Q | PC/PI | 82 | 83 |  |  | No Q are P | Some M are Q | PC/PI | 43 | 71 |
|  |  |  | No M are Q | PC/PI | 17 | 55 |  |  |  | No M are Q | PC/PI | 21 | 68 |
|  |  |  | Some M are not Q | PC/PI | 78 | 81 |  |  |  | Some M are not Q | PC/PI | 54 | 78 |
| 2 | Q - M | Some P are not M | All Q are M | PC/PI | 4 | 43 | 2 | Q - M | Some P are not M | All Q are M | PC/PI | 9 | 27 |
|  |  | All Q are P | Some Q are M | PC/PI | 57 | 85 |  |  | No Q are P | Some Q are M | PC/PI | 47 | 89 |
|  |  |  | No Q are M | PC/PI | 25 | 58 |  |  |  | No Q are M | PC/PI | 9 | 31 |
|  |  |  | Some Q are not M | PC/PI | 86 | 89 |  |  |  | Some Q are not M | PC/PI | 47 | 94 |
| 2 | M - Q | Some P are not M | All M are Q | PC/PI | 8 | 35 | 2 | M - Q | Some P are not M | All M are Q | PC/PI | 4 | 28 |
|  |  | Some Q are P | Some M are Q | PC/PI | 62 | 86 |  |  | Some Q are not P | Some M are Q | PC/PI | 82 | 90 |
|  |  |  | No M are Q | PC/PI | 9 | 43 |  |  |  | No M are Q | PC/PI | 16 | 47 |
|  |  |  | Some M are not Q | PC/PI | 71 | 96 |  |  |  | Some M are not Q | PC/PI | 82 | 92 |
| 2 | Q - M | Some P are not M | All Q are M | PC/PI | 9 | 32 | 2 | Q - M | Some P are not M | All Q are M | PC/PI | 0 | 24 |
|  |  | Some Q are P | Some Q are M | PC/PI | 57 | 92 |  |  | Some Q are not P | Some Q are M | PC/PI | 65 | 99 |
|  |  |  | No Q are M | PC/PI | 19 | 47 |  |  |  | No Q are M | PC/PI | 8 | 53 |
|  |  |  | Some Q are not M | PC/PI | 81 | 88 |  |  |  | Some Q are not M | PC/PI | 78 | 87 |

**Appendix 2 (to be continue)**

| **S** | **Inf.**  **order** | **propositions** | **inferences** | **AL** | **□** | **◊** | **S** | **Inf.**  **order** | **propositions** | **inferences** | **AL** | **□** | **◊** |
| --- | --- | --- | --- | --- | --- | --- | --- | --- | --- | --- | --- | --- | --- |
| 3 | M - Q | All M are P | All M are Q | PC/PI | 58 | 80 | 3 | M - Q | All M are P | All M are Q | IPI | 5 | 9 |
|  |  | All Q are P | Some M are Q | PC/PI | 25 | 65 |  |  | No Q are P | Some M are Q | IPI | 9 | 23 |
|  |  |  | No M are Q | PC/PI | 19 | 22 |  |  |  | No M are Q | NC | 84 | 90 |
|  |  |  | Some M are not Q | PC/PI | 21 | 33 |  |  |  | Some M are not Q | NC | 36 | 79 |
| 3 | Q - M | All M are P | All Q are M | PC/PI | 64 | 75 | 3 | Q - M | All M are P | All Q are M | IPI | 9 | 14 |
|  |  | All Q are P | Some Q are M | PC/PI | 45 | 63 |  |  | No Q are P | Some Q are M | IPI | 8 | 28 |
|  |  |  | No Q are M | PC/PI | 19 | 26 |  |  |  | No Q are M | NC | 83 | 80 |
|  |  |  | Some Q are not M | PC/PI | 20 | 33 |  |  |  | Some Q are not M | NC | 43 | 63 |
| 3 | M - Q | All M are P | All M are Q | PC/PI | 4 | 28 | 3 | M - Q | All M are P | All M are Q | PC/PI | 19 | 41 |
|  |  | Some Q are P | Some M are Q | PC/PI | 64 | 72 |  |  | Some Q are not P | Some M are Q | PC/PI | 84 | 87 |
|  |  |  | No M are Q | PC/PI | 21 | 50 |  |  |  | No M are Q | PC/PI | 44 | 56 |
|  |  |  | Some M are not Q | PC/PI | 83 | 89 |  |  |  | Some M are not Q | PC/PI | 90 | 91 |
| 3 | Q - M | All M are P | All Q are M | PC/PI | 24 | 34 | 3 | Q - M | All M are P | All Q are M | IPI | 0 | 9 |
|  |  | Some Q are P | Some Q are M | PC/PI | 69 | 82 |  |  | Some Q are not P | Some Q are M | PC/PI | 37 | 67 |
|  |  |  | No Q are M | PC/PI | 11 | 29 |  |  |  | No Q are M | PC/PI | 39 | 80 |
|  |  |  | Some Q are not M | PC/PI | 81 | 95 |  |  |  | Some Q are not M | NC | 83 | 93 |
| 3 | M - Q | Some M are P | All M are Q | PC/PI | 0 | 35 | 3 | M - Q | Some M are P | All M are Q | IPI | 11 | 15 |
|  |  | All Q are P | Some M are Q | PC/PI | 68 | 80 |  |  | No Q are P | Some M are Q | PC/PI | 35 | 68 |
|  |  |  | No M are Q | PC/PI | 4 | 45 |  |  |  | No M are Q | PC/PI | 44 | 79 |
|  |  |  | Some M are not Q | PC/PI | 73 | 89 |  |  |  | Some M are not Q | NC | 82 | 88 |
| 3 | Q - M | Some M are P | All Q are M | PC/PI | 15 | 29 | 3 | Q - M | Some M are P | All Q are M | PC/PI | 0 | 19 |
|  |  | All Q are P | Some Q are M | PC/PI | 55 | 85 |  |  | No Q are P | Some Q are M | PC/PI | 36 | 47 |
|  |  |  | No Q are M | PC/PI | 4 | 41 |  |  |  | No Q are M | PC/PI | 46 | 81 |
|  |  |  | Some Q are not M | PC/PI | 58 | 88 |  |  |  | Some Q are not M | PC/PI | 47 | 81 |
| 3 | M - Q | Some M are P | All M are Q | PC/PI | 12 | 31 | 3 | M - Q | Some M are P | All M are Q | PC/PI | 9 | 18 |
|  |  | Some Q are P | Some M are Q | PC/PI | 65 | 91 |  |  | Some Q are not P | Some M are Q | PC/PI | 61 | 92 |
|  |  |  | No M are Q | PC/PI | 9 | 51 |  |  |  | No M are Q | PC/PI | 15 | 58 |
|  |  |  | Some M are not Q | PC/PI | 61 | 90 |  |  |  | Some M are not Q | PC/PI | 71 | 91 |
| 3 | Q - M | Some M are P | All Q are M | PC/PI | 11 | 11 | 3 | Q - M | Some M are P | All Q are M | PC/PI | 5 | 33 |
|  |  | Some Q are P | Some Q are M | PC/PI | 19 | 22 |  |  | Some Q are not P | Some Q are M | PC/PI | 62 | 89 |
|  |  |  | No Q are M | PC/PI | 79 | 92 |  |  |  | No Q are M | PC/PI | 9 | 54 |
|  |  |  | Some Q are not M | PC/PI | 34 | 71 |  |  |  | Some Q are not M | PC/PI | 77 | 85 |
| 3 | M - Q | No M are P | All M are Q | IPI | 4 | 15 | 3 | M - Q | No M are P | All M are Q | PC/PI | 14 | 67 |
|  |  | All Q are P | Some M are Q | IPI | 9 | 15 |  |  | No Q are P | Some M are Q | PC/PI | 29 | 92 |
|  |  |  | No M are Q | NC | 81 | 85 |  |  |  | No M are Q | PC/PI | 49 | 69 |
|  |  |  | Some M are not Q | NC | 42 | 55 |  |  |  | Some M are not Q | PC/PI | 8 | 70 |
| 3 | Q - M | No M are P | All Q are M | IPI | 45 | 83 | 3 | Q - M | No M are P | All Q are M | PC/PI | 7 | 27 |
|  |  | All Q are P | Some Q are M | IPI | 61 | 78 |  |  | No Q are P | Some Q are M | PC/PI | 21 | 29 |
|  |  |  | No Q are M | NC | 9 | 4 |  |  |  | No Q are M | PC/PI | 44 | 78 |
|  |  |  | Some Q are not M | NC | 35 | 58 |  |  |  | Some Q are not M | PC/PI | 22 | 53 |
| 3 | M - Q | No M are P | All M are Q | PC/PI | 60 | 82 | 3 | M - Q | No M are not P | All M are Q | PC/PI | 15 | 30 |
|  |  | Some Q are P | Some M are Q | PC/PI | 4 | 32 |  |  | Some Q are not P | Some M are Q | PC/PI | 30 | 47 |
|  |  |  | No M are Q | PC/PI | 77 | 84 |  |  |  | No M are Q | PC/PI | 59 | 88 |
|  |  |  | Some M are not Q | PC/PI | 85 | 90 |  |  |  | Some M are not Q | PC/PI | 33 | 54 |
| 3 | Q - M | No M are P | All Q are M | IPI | 7 | 23 | 3 | Q - M | No M are not P | All Q are M | PC/PI | 4 | 11 |
|  |  | Some Q are P | Some Q are M | PC/PI | 80 | 9 |  |  | Some Q are not P | Some Q are M | PC/PI | 56 | 81 |
|  |  |  | No Q are M | PC/PI | 9 | 41 |  |  |  | No Q are M | PC/PI | 30 | 58 |
|  |  |  | Some Q are not M | NC | 88 | 89 |  |  |  | Some Q are not M | PC/PI | 52 | 78 |
| 3 | M - Q | Some M are not P | All M are Q | IPI | 9 | 41 | 3 | M - Q | Some M are not P | All M are Q | PC/PI | 4 | 16 |
|  |  | All Q are P | Some M are Q | PC/PI | 82 | 92 |  |  | No Q are P | Some M are Q | PC/PI | 35 | 67 |
|  |  |  | No M are Q | PC/PI | 7 | 35 |  |  |  | No M are Q | PC/PI | 21 | 68 |
|  |  |  | Some M are not Q | NC | 87 | 91 |  |  |  | Some M are not Q | PC/PI | 54 | 78 |
| 3 | Q - M | Some M are not P | All Q are M | PC/PI | 4 | 43 | 3 | Q - M | Some M are not P | All Q are M | PC/PI | 59 | 79 |
|  |  | All Q are P | Some Q are M | PC/PI | 57 | 85 |  |  | No Q are P | Some Q are M | PC/PI | 47 | 89 |
|  |  |  | No Q are M | PC/PI | 25 | 58 |  |  |  | No Q are M | PC/PI | 29 | 71 |
|  |  |  | Some Q are not M | PC/PI | 86 | 89 |  |  |  | Some Q are not M | PC/PI | 67 | 94 |
| 3 | M - Q | Some M are not P | All M are Q | PC/PI | 8 | 35 | 3 | M - Q | Some M are not P | All M are Q | PC/PI | 14 | 38 |
|  |  | Some Q are P | Some M are Q | PC/PI | 62 | 86 |  |  | Some Q are not P | Some M are Q | PC/PI | 85 | 92 |
|  |  |  | No M are Q | PC/PI | 9 | 43 |  |  |  | No M are Q | PC/PI | 55 | 57 |
|  |  |  | Some M are not Q | PC/PI | 81 | 96 |  |  |  | Some M are not Q | PC/PI | 62 | 100 |
| 3 | Q - M | Some M are not P | All Q are M | PC/PI | 4 | 29 | 3 | Q - M | Some M are not P | All Q are M | PC/PI | 4 | 46 |
|  |  | Some Q are P | Some Q are M | PC/PI | 62 | 95 |  |  | Some Q are not P | Some Q are M | PC/PI | 51 | 100 |
|  |  |  | No Q are M | PC/PI | 19 | 40 |  |  |  | No Q are M | PC/PI | 18 | 44 |
|  |  |  | Some Q are not M | PC/PI | 72 | 92 |  |  |  | Some Q are not M | PC/PI | 68 | 82 |

**Appendix 2 (to be continue)**

| **S** | **Inf.**  **order** | **propositions** | **inferences** | **AL** | **□** | **◊** | **S** | **Inf.**  **order** | **propositions** | **inferences** | **AL** | **□** | **◊** |
| --- | --- | --- | --- | --- | --- | --- | --- | --- | --- | --- | --- | --- | --- |
| 4 | M - Q | All P are M | All M are Q | PC/PI | 58 | 80 | 4 | M - Q | All P are M | All M are Q | IPI | 9 | 21 |
|  |  | All P are Q | Some M are Q | NC | 25 | 65 |  |  | No P are Q | Some M are Q | IPI | 25 | 22 |
|  |  |  | No M are Q | IPI | 19 | 22 |  |  |  | No M are Q | NC | 64 | 94 |
|  |  |  | Some M are not Q | PC/PI | 21 | 33 |  |  |  | Some M are not Q | NC | 39 | 78 |
| 4 | Q - M | All P are M | All Q are M | PC/PI | 64 | 75 | 4 | Q - M | All P are M | All Q are M | PC/PI | 9 | 13 |
|  |  | All P are Q | Some Q are M | NC I | 45 | 63 |  |  | No P are Q | Some Q are M | PC/PI | 18 | 28 |
|  |  |  | No Q are M | PC/PI | 19 | 26 |  |  |  | No Q are M | PC/PI | 83 | 91 |
|  |  |  | Some Q are not M | PC/PI | 20 | 33 |  |  |  | Some Q are not M | PC/PI | 43 | 67 |
| 4 | M - Q | All P are M | All M are Q | PC/PI | 4 | 28 | 4 | M - Q | All P are M | All M are Q | IPI | 9 | 39 |
|  |  | Some P are Q | Some M are Q | NC | 64 | 72 |  |  | Some P are not Q | Some M are Q | PC/PI | 74 | 87 |
|  |  |  | No M are Q | IPI | 21 | 50 |  |  |  | No M are Q | PC/PI | 24 | 52 |
|  |  |  | Some M are not Q | PC/PI | 83 | 89 |  |  |  | Some M are not Q | NC | 84 | 89 |
| 4 | Q - M | All P are M | All Q are M | PC/PI | 24 | 34 | 4 | Q - M | All P are M | All Q are M | PC/PI | 0 | 21 |
|  |  | Some P are Q | Some Q are M | NC | 69 | 82 |  |  | Some P are not Q | Some Q are M | PC/PI | 57 | 87 |
|  |  |  | No Q are M | PC/PI | 11 | 29 |  |  |  | No Q are M | PC/PI | 17 | 29 |
|  |  |  | Some Q are not M | PC/PI | 81 | 95 |  |  |  | Some Q are not M | PC/PI | 76 | 82 |
| 4 | M - Q | Some P are M | All M are Q | PC/PI | 0 | 35 | 4 | M - Q | Some P are M | All M are Q | IPI | 9 | 25 |
|  |  | All P are Q | Some M are Q | PC/PI | 68 | 80 |  |  | No P are Q | Some M are Q | PC/PI | 37 | 62 |
|  |  |  | No M are Q | PC/PI | 4 | 45 |  |  |  | No M are Q | PC/PI | 40 | 81 |
|  |  |  | Some M are not Q | NC | 73 | 89 |  |  |  | Some M are not Q | NC | 72 | 86 |
| 4 | Q - M | Some P are M | All Q are M | PC/PI | 15 | 29 | 4 | Q - M | Some P are M | All Q are M | PC/PI | 9 | 19 |
|  |  | All P are Q | Some Q are M | NC | 55 | 85 |  |  | No P are Q | Some Q are M | PC/PI | 76 | 67 |
|  |  |  | No Q are M | IPI | 4 | 41 |  |  |  | No Q are M | PC/PI | 9 | 47 |
|  |  |  | Some Q are not M | PC/PI | 58 | 88 |  |  |  | Some Q are not M | PC/PI | 79 | 91 |
| 4 | M - Q | Some P are M | All M are Q | PC/PI | 12 | 31 | 4 | M - Q | Some P are M | All M are Q | PC/PI | 0 | 28 |
|  |  | Some P are Q | Some M are Q | PC/PI | 65 | 91 |  |  | Some P are not Q | Some M are Q | PC/PI | 59 | 87 |
|  |  |  | No M are Q | PC/PI | 9 | 51 |  |  |  | No M are Q | PC/PI | 15 | 58 |
|  |  |  | Some M are not Q | PC/PI | 61 | 90 |  |  |  | Some M are not Q | PC/PI | 69 | 91 |
| 4 | Q - M | Some P are M | All Q are M | PC/PI | 11 | 11 | 4 | Q - M | Some P are M | All Q are M | PC/PI | 25 | 53 |
|  |  | Some P are Q | Some Q are M | PC/PI | 19 | 22 |  |  | Some P are not Q | Some Q are M | PC/PI | 62 | 89 |
|  |  |  | No Q are M | PC/PI | 79 | 92 |  |  |  | No Q are M | PC/PI | 69 | 84 |
|  |  |  | Some Q are not M | PC/PI | 34 | 71 |  |  |  | Some Q are not M | PC/PI | 77 | 85 |
| 4 | M - Q | No P are M | All M are Q | PC/PI | 4 | 15 | 4 | M - Q | No P are M | All M are Q | PC/PI | 26 | 67 |
|  |  | All P are Q | Some M are Q | PC/PI | 9 | 15 |  |  | No P are Q | Some M are Q | PC/PI | 19 | 52 |
|  |  |  | No M are Q | PC/PI | 81 | 85 |  |  |  | No M are Q | PC/PI | 44 | 82 |
|  |  |  | Some M are not Q | PC/PI | 42 | 55 |  |  |  | Some M are not Q | PC/PI | 8 | 70 |
| 4 | Q - M | No P are M | All Q are M | PC/PI | 45 | 83 | 4 | Q - M | No P are M | All Q are M | PC/PI | 7 | 27 |
|  |  | All P are Q | Some Q are M | PC/PI | 61 | 78 |  |  | No P are Q | Some Q are M | PC/PI | 21 | 29 |
|  |  |  | No Q are M | PC/PI | 9 | 4 |  |  |  | No Q are M | PC/PI | 44 | 78 |
|  |  |  | Some Q are not M | PC/PI | 35 | 58 |  |  |  | Some Q are not M | PC/PI | 22 | 53 |
| 4 | M - Q | No P are M | All M are Q | PC/PI | 60 | 82 | 4 | M - Q | No P are not M | All M are Q | PC/PI | 15 | 30 |
|  |  | Some P are Q | Some M are Q | PC/PI | 4 | 32 |  |  | Some P are not Q | Some M are Q | PC/PI | 30 | 47 |
|  |  |  | No M are Q | PC/PI | 77 | 84 |  |  |  | No M are Q | PC/PI | 59 | 88 |
|  |  |  | Some M are not Q | PC/PI | 85 | 90 |  |  |  | Some M are not Q | PC/PI | 33 | 54 |
| 4 | Q - M | No P are M | All Q are M | IPI | 7 | 23 | 4 | Q - M | No P are not M | All Q are M | PC/PI | 4 | 11 |
|  |  | Some P are Q | Some Q are M | PC/PI | 80 | 9 |  |  | Some P are not Q | Some Q are M | PC/PI | 56 | 81 |
|  |  |  | No Q are M | PC/PI | 9 | 41 |  |  |  | No Q are M | PC/PI | 30 | 58 |
|  |  |  | Some Q are not M | NC | 88 | 89 |  |  |  | Some Q are not M | PC/PI | 52 | 78 |
| 4 | M - Q | Some P are not M | All M are Q | PC/PI | 9 | 41 | 4 | M - Q | Some P are not M | All M are Q | PC/PI | 4 | 16 |
|  |  | All P are Q | Some M are Q | PC/PI | 82 | 92 |  |  | No P are Q | Some M are Q | PC/PI | 35 | 67 |
|  |  |  | No M are Q | PC/PI | 7 | 35 |  |  |  | No M are Q | PC/PI | 21 | 68 |
|  |  |  | Some M are not Q | PC/PI | 87 | 91 |  |  |  | Some M are not Q | PC/PI | 54 | 78 |
| 4 | Q - M | Some P are not M | All Q are M | IPI | 4 | 43 | 4 | Q - M | Some P are not M | All Q are M | PC/PI | 19 | 29 |
|  |  | All P are Q | Some Q are M | PC/PI | 57 | 85 |  |  | No P are Q | Some Q are M | PC/PI | 47 | 89 |
|  |  |  | No Q are M | PC/PI | 25 | 58 |  |  |  | No Q are M | PC/PI | 39 | 71 |
|  |  |  | Some Q are not M | NC | 86 | 89 |  |  |  | Some Q are not M | PC/PI | 49 | 87 |
| 4 | M - Q | Some P are not M | All M are Q | PC/PI | 8 | 35 | 4 | M - Q | Some P are not M | All M are Q | PC/PI | 17 | 38 |
|  |  | Some P are Q | Some M are Q | PC/PI | 62 | 86 |  |  | Some P are not Q | Some M are Q | PC/PI | 65 | 92 |
|  |  |  | No M are Q | PC/PI | 9 | 43 |  |  |  | No M are Q | PC/PI | 55 | 57 |
|  |  |  | Some M are not Q | PC/PI | 81 | 96 |  |  |  | Some M are not Q | PC/PI | 59 | 97 |
| 4 | Q - M | Some P are not M | All Q are M | PC/PI | 4 | 29 | 4 | Q - M | Some P are not M | All Q are M | PC/PI | 4 | 42 |
|  |  | Some P are Q | Some Q are M | PC/PI | 62 | 95 |  |  | Some P are not Q | Some Q are M | PC/PI | 51 | 96 |
|  |  |  | No Q are M | PC/PI | 19 | 40 |  |  |  | No Q are M | PC/PI | 17 | 54 |
|  |  |  | Some Q are not M | PC/PI | 72 | 92 |  |  |  | Some Q are not M | PC/PI | 67 | 93 |

S.= Shape; Inf. Order= inference order; AL = Alethic Logic; □ = Necessity; ◊ = Possibility; NC = Necessary Consistency; IPI = Impossible incompatibility; PC/PI = Possible Consistency and Possible Incompatibility. Here, M denotes ♦️, P denotes ▱, and Q denotes🪁.
